# Measuring impact of protected area management interventions: current and future use of the Global Database of Protected Area Management Effectiveness

**DOI:** 10.1098/rstb.2014.0281

**Published:** 2015-11-05

**Authors:** Lauren Coad, Fiona Leverington, Kathryn Knights, Jonas Geldmann, April Eassom, Valerie Kapos, Naomi Kingston, Marcelo de Lima, Camilo Zamora, Ivon Cuardros, Christoph Nolte, Neil D. Burgess, Marc Hockings

**Affiliations:** 1 Environmental Change Institute, Oxford University Centre for the Environment, South Parks Road, Oxford OX1 3QY, UK; 2 United Nations Environment Programme World Conservation Monitoring Centre, 219 Huntingdon Road, Cambridge CB3 0DL, UK; 3 University of Queensland, St Lucia, QLD 4072, Queensland, Australia; 4 International Union for Conservation of Nature Global Protected Areas Programme (IUCN-WCPA), Rue Mauverney 28, 1196, Gland, Switzerland; 5 Protected Area Solutions, 38 Foothill Place, The Gap, QLD 4061, Queensland, Australia; 6 Center for Macroecology, Evolution and Climate, University of Copenhagen, DK-2100 Copenhagen, Denmark; 7 International Forestry Resources and Institutions, School of Natural Resources and Environment, University of Michigan, 440 Church Street, Ann Arbor, MI 48109-1041, USA

**Keywords:** conservation outcomes, impact assessment, biodiversity, convention on biological diversity (CBD), biodiversity targets

## Abstract

Protected areas (PAs) are at the forefront of conservation efforts, and yet despite considerable progress towards the global target of having 17% of the world's land area within protected areas by 2020, biodiversity continues to decline. The discrepancy between increasing PA coverage and negative biodiversity trends has resulted in renewed efforts to enhance PA effectiveness. The global conservation community has conducted thousands of assessments of protected area management effectiveness (PAME), and interest in the use of these data to help measure the conservation impact of PA management interventions is high. Here, we summarize the status of PAME assessment, review the published evidence for a link between PAME assessment results and the conservation impacts of PAs, and discuss the limitations and future use of PAME data in measuring the impact of PA management interventions on conservation outcomes. We conclude that PAME data, while designed as a tool for local adaptive management, may also help to provide insights into the impact of PA management interventions from the local-to-global scale. However, the subjective and ordinal characteristics of the data present significant limitations for their application in rigorous scientific impact evaluations, a problem that should be recognized and mitigated where possible.

## 1. Introduction

The convention on biological diversity (CBD) calls for the protection of 17% of terrestrial area and 10% of the world's oceans, through effectively and equitably managed, ecologically representative and well-connected systems of protected areas (PAs) and other effective area-based conservation measures, by 2020 (Aichi Target 11 [1]). Although PA coverage is approaching these percentage targets in many parts of the terrestrial and marine realms [2,3], global biodiversity is still declining [4]. The fact that declines are also seen within many PAs [5,6] emphasizes the need to enhance the effectiveness of existing PAs for conserving biodiversity [7], in addition to increasing the area under protection [8,9].

Our understanding of the impact of PA management interventions on conservation outcomes has been impeded by a lack of data [6]. The World Database on PAs [10] contains basic PA characteristics such as location, size and management category reported by national governments for more than 200 000 sites [3]. However, the collection of data on individual management actions, outputs and outcomes of PAs, and outcomes at matched, unprotected sites, which are required for understanding PA impact, depends on reporting at the site level, and is therefore very resource-intensive. The need for national and regional datasets on PA management is reflected in conservation policy; the CBD calls for: *‘* *…* Parties to *…* expand and institutionalize management effectiveness assessments to work towards assessing 60 per cent of the total area of PAs by 2015 using various national and regional tools, and report the results into the global database on management effectiveness…’ (CBD Programme of Work on Protected Areas (PoWPA)) [11, p. 5].

Since the mid-1990s, various methodologies have been developed for assessing PA management effectiveness (PAME) [12–14]. Assessment data from all over the world have now been collated in the Global Database for Protected Area Management Effectiveness (GD-PAME; electronic supplementary material, S1), which is summarized in this paper and currently contains records of almost 18 000 PAME assessments—the only global dataset on PA management. The GD-PAME includes information about the methodologies and indicators used, and records details of individual assessments. It also reports PAME results under a set of headline indicators (electronic supplementary material, S2), standardizing data from a wide range of methodologies [14].

PAME assessments were originally developed to support adaptive management of PAs at site level and system level. Their primary purposes were (i) to improve PA management through information sharing and adaptive management; (ii) to more effectively allocate resources to the PAs or the management themes most in need (e.g. shifting attention to law enforcement or pest plant management); (iii) to provide accountability and reporting at local, national or international levels; and (iv) to increase community awareness of PA management and issues.

The PAME assessments hold a wealth of information on PA management efforts. They have been applied globally, and use standardized methods for collecting management data. Given this, the collated dataset provides a potentially valuable resource for measuring and understanding the impacts of PA management interventions. Impact evaluation aims to understand the intended and unintended impacts that are caused by an intervention [15], in this case, the impact of PA management on biodiversity outcomes. Rigorous scientific impact evaluation [16] requires a comparison of observed PA outcomes in the presence of the management intervention with the counterfactual, i.e. the outcomes that would have occurred in the absence of the intervention. Counterfactuals can be determined using either an experimental or semi-experimental study design [17]. In general, PAME methodologies collect neither quantitative data on biodiversity outcomes nor counterfactual data. However, PA impact can be measured by applying quasi-experimental methods to independently collected biodiversity data, and PAME variables can then be used to investigate correlations associated with the observed effect sizes. Analyses might test hypotheses that improvements in PA management will result in improvements in observed outcomes relative to the counterfactual; for example, increases in PAME scores might correlate with recovery of species populations that are declining outside PAs. As an initial assessment of the strengths and weaknesses of PAME datasets for evaluating the impact of PA management on biodiversity outcomes, we therefore:

(1) discuss the conceptual roots of PAME and to what extent understanding management impacts forms part of the rationale for the development of PAME;
(2) explore the different types of PAME assessment, including how PAME methodologies capture information on PA management and biodiversity outcomes, and the users and uses of PAME to date;
(3) present available PAME datasets and discuss the regional and ecological coverage of these data, measuring progress towards the CBD PAME targets;
(4) review the available evidence for a link between PAME scores and PA management outcomes and impacts; and
(5) identify limitations and propose approaches for overcoming them.

To standardize our use of terminology, we use the monitoring and evaluation definitions of Mascia *et al.* [15].

## 2. Conceptual roots of protected area management effectiveness

Conceptually, most PAME approaches relate to the International Union for Conservation of Nature World Commission on Protected Areas (IUCN WCPA) framework [18], which is based on the theory of change (ToC) [19] and the logic model framework [20]. With these frameworks, evaluators construct a model of reality, using pictures or words to understand the relationships among the resources they have to operate their programme, the activities they plan and the changes or results they hope to achieve. PAME evaluations also have affinity to the utilization-based evaluation approach [21], which focuses on improving management rather than on gathering data for its own sake. An important aspect of this philosophy is the recognition that the process of evaluation can itself bring about improvements in practice. PAME methodologies are therefore designed to capture information on management elements hypothesized to contribute to biodiversity outcomes.

PAME assessments evaluate the management elements of planning, inputs, processes, outputs and outcomes within an assessed context. These elements form a results chain similar to that of logic models, but are more often depicted as steps around a management cycle (electronic supplementary material, S3), to stress the feedback of information into an adaptive management process. In an overview of approaches to conservation monitoring and evaluation, Mascia *et al.* [15] suggest that questionnaire-based PAME assessments should be regarded as management assessments, which have a focal question of ‘What are the management inputs, activities, and outputs associated with a conservation intervention, and how are these changing over time?’ However, IUCN guidance on PAME [18, p. 39] stresses that ‘an important part of the analysis of PAME data should be to identify the extent to which measured outcomes are due to management interventions or to other factors, which may be beyond a manager's control […] It is important to understand the causes of success or failure of management: without such an analysis, attempts to improve performance may be ineffective’. The rationale for PAME, while focused on facilitating effective management rather than building a scientific evidence base, is therefore, in part, to understand the impacts of PA management.

## 3. Available protected area management effectiveness data and methodologies

**Figure 1.**
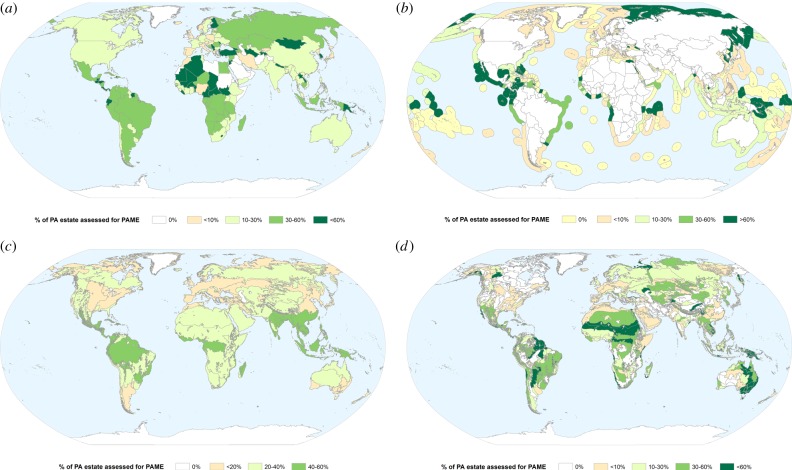
Progress towards the 60% PAME assessment target of the CBD Programme of Work on Protected Areas, by (*a*) terrestrial territory of countries, (*b*) marine territory of countries, (*c*) WWF biomes and (*d*) WWF terrestrial ecoregions.

As of January 2015, 17 739 PAME assessments had been collated in the GD-PAME, representing 9037 PAs, with 3666 sites having multiple assessments. Some 17.5% of countries have already met the CBD PoWPA 2015 60% PAME assessment target (figure 1*a,b*; methods in electronic supplementary material, S4). Among major biomes (figure 1*c* and electronic supplementary material, S4) and ecoregions (figure 1*d* and electronic supplementary material, S4) [22], the frequency of PAME assessment is highest in the tropical forests, where 45% of PAs have been assessed. The PoWPA target has been met for over 16% of all ecoregions (and 14% of the WWF Global 200 ecoregions identified by WWF as priorities for conservation).

Ninety-five PAME assessment methodologies are recorded in the GD-PAME [12–14]. Most are self-assessment scorecards that contain a number of questions scored against a Likert-type [23] or ordinal scales to measure progress towards specific management standards, such as the existence of a management plan or the adequacy of law enforcement activities. They also record quantitative information, such as PA budget and staff numbers, and information on perceived PA objectives and threats. The most commonly used PAME tools contain few questions relating to biological or social outcomes (table 1). PAME assessments are often completed over 1–3 days by a group of PA stakeholders, who may include the PA managers and partners and sometimes representatives from local government, local communities and NGOs.

**Table 1.** A summary of the management information and outcomes data collected by four widely used PAME methodologies. Further details on each methodology are available in electronic supplementary material, S7.

attributes	management effectiveness tracking tool (METT)	rapid assessment and prioritization of protected area management (RAPPAM)	enhancing our heritage (EOH)	state of the parks (SOP)
overall structure of methodology	rapid assessment scorecard of 30 questions across all six IUCN-WCPA elements but with emphasis on context, planning, inputs, and processes. Also collects information on budgets, staffing, principal PA values, objectives, and threats	designed for broad-level comparisons among many PAs that together make a PAs network or system. It covers five of the WCPA management effectiveness elements (context, planning, inputs, processes and outputs)	workbook of 12 tools based on all six IUCN-WCPA elements. The tools identify main site values (biodiversity, social, economic and cultural), assessing whether appropriate objectives based on these values have been set, and then evaluating the effectiveness of management in achieving these objectives	a proforma that addresses each of the six elements of the IUCN-WCPA Framework. The proforma is designed to be completed for all or most PAs in a system. It incorporates both quantitative and qualitative assessment items with information values, threats and stakeholders, resourcing and planning, and 30 items assessing management performance and outcomes
data types	lists of principal values and assessment of extent of nominated threats. Scorecard records performance on four-point ordinal scale using descriptions of management performance, where 3 describes an ideal situation and 0 represents very poor or no performance	most questions use a standard 4-point scale (no = 0, mostly no = 1, mostly yes = 3, yes = 5), where ‘yes' describes an ideal situation. Threats (vulnerability) are rated according to their extent, impact and trend	mix of quantitative, qualitative, and scoring data. Identified key values and threats are used to design monitoring programmes to provide quantitative data on condition. Includes collection of information on sources of evidence for assessments	mix of quantitative, qualitative and scoring data. Includes collection of information on sources of evidence for assessments
methodology implementation	usually done in less than a day with input from managers and project staff	workshop format (1–2 days) with managers and other knowledgeable participants (e.g. Agency and NGO staff, scientists) across the range of PAs involved in the assessment	compilation of data from monitoring and other sources. Mix of workshops with staff and stakeholders, scientists and community representatives and preparation of assessment report by project staff, usually over a period of a few months	usually undertaken in workshop (1 day) by managers and other specialist staff. Follow-up audit and validation of data by central agency staff working with assessors as needed
outcomes data	one indicator on biological outcomes	none	indicators for status and trend of key values defined for each site	indicators for outcomes related to each status and trend of biological and cultural values and threats, with optional more detailed information by species
counterfactual data (whether outcomes can be attributed to management)	none	none	none	includes some counterfactual assessment asking evaluators whether changes can be attributed to management actions or external causes

The most widely used PAME methodologies are the management effectiveness tracking tool (METT) [24] (completed 4046 times for 2045 PAs, with repeat assessments conducted over different years available for 833 PAs); the New South Wales State of Our Parks (SOP) methodology [25] (3552 times, 859 PAs, 764 repeats); and the Rapid Assessments and Prioritization of Protected Area Management (RAPPAM) [26] (2276 times, 1930 PAs, 322 repeats). For further details, see electronic supplementary material, S5.

## 4. Users and uses of protected area management effectiveness methodologies

**Figure 2.**
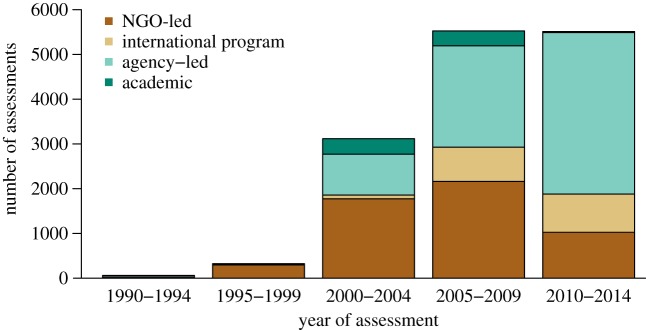
Application of PAME tools from 1990 to 2014, according to the implementing organization.

There has been a sustained increase in PAME assessments since 1990, and three distinct periods of application are identified (figure 2). Until the mid-2000s, the development and early adoption of the methodologies were primarily by NGOs—especially WWF, who helped develop the METT and RAPPAM tools to track progress towards goals in improving PA management [24,27,28].

Subsequently, PAME was adopted as a performance evaluation tool by funding agencies, such as the global environment facility (GEF), with large PA project portfolios across many countries [29]. The GEF requires that three METT assessments be conducted in all funded PAs over the course of a GEF project, and project performance is then measured in terms of the change in overall METT score from baseline to final assessment. The GEF makes the assumption that improvements in PA management owing to project interventions will have a positive impact on biodiversity, based on commissioned studies which found that increases in PAME scores were correlated with improvements in biodiversity outcomes [30,31].

The past decade has seen an increase in assessments led by national PA agencies and government ministries (e.g. New South Wales and Victoria, Australia [32,33], Colombia, South Korea [34] Finland [35] and Indonesia [36]), largely driven by agency objectives to implement an adaptive management approach, improve planning and priority setting, and report on the status of PA management at a national level [25]. This may also reflect the requirement for countries to report to the CBD on Aichi Target 11 [1].

## 5. Evidence for correlations between protected area management effectiveness scores and protected area outcomes and impacts

To assess how PAME data have been used in impact evaluation, we reviewed the peer-reviewed literature (search methodology and search terms; electronic supplementary material, S6) to assess the current knowledge on the correlations between PA management, as measured using PAME data, and biodiversity outcomes measured by external methods. We reviewed 185 papers at the level of title and abstract, retaining 23 papers for detailed review of the main text, which yielded a final set of five peer-reviewed papers. Papers excluded at both stages were (i) papers that did not employ a PAME methodology; (ii) those that did not relate PAME results to a measure of outcome or impact owing to the PA. Individual reasons for exclusion of papers at the second-review stage are given in electronic supplementary material, S6. In addition, four ‘grey literature’ reports were identified by searching online libraries and web pages of agencies and organizations known to use PAME assessment tools. This gave a total of nine studies for further analysis, all from tropical developing countries, mostly in Africa and Latin America (table 2).

**Table 2.** Studies that have investigated the relationship between PA management quality (using PAME tools) and conservation outcomes and impact.

study	location	PAME type	sample size (PAs)	use of PAME data (predictor variable)	counterfactual methodology?	outcome measure	direction of relationship
Carranza *et al.* [37]	Brazil	RAPPAM	26	total score and individual scores	Y	change in forest cover	0
Henschel *et al.* [38]	West Africa	METT	12	budget and staffing + total score for 13 selected questions	N	differences in lion (*Panthera leo*) presence	+
Great Barrier Reef Marine Park Authority [39]	The Great Barrier Reef (Australia)	GBR Outlook	1	management effectiveness assessment by thematic area (e.g. water quality, tourism)	N	change in condition of outcomes for thematic areas	+
Mwangi *et al.* [40]	Kenya	BirdLife evaluation	36	total score	N	change in condition of the key habitats for Important Bird Area trigger species	0
Nolte & Agrawal [41]	Amazon Basin (Brazil, Bolivia, and Peru)	METT	41	total score	Y	changes in fire frequency between PAs	0
Nolte *et al.* [42]	Brazil	RAPPAM	66	total score	Y	change in forest cover	0
Walker & Walker [43]	Belize	NPAPSP**	72	total score	N	expert-assessed biodiversity indicator	+
Zimsky *et al.* [44]	Zambia	METT	11	change in total score	N	changes in population sizes of tigers, leopards, spotted deer, Sambar deer, gaurs	+
Zimsky *et al.* [30]	India	METT	7	change in total score	N	expert-based assessment of species (not specified)	+

**National Protected Areas Policy and Systems Plan, PAME System designed for Belize protected areas. (+) = positive correlation between management effectiveness and conservation outcomes. (0) = no correlation detected between management effectiveness and conservation outcomes. NPAPSP, National Protected Areas Policy and Systems Plan.

Three studies used remotely sensed data on tree cover change to measure the impact of PAs on deforestation rate, using statistical matching methods to construct counterfactuals, and then investigated correlations between PAME scores and the size of the measured impacts [6–8]. The remaining six studies measured correlations between PAME and biodiversity outcomes, using data collected *in situ* on changes in animal populations, species distributions or expert assessments of the state of biodiversity. Six of the nine studies focused on overall management performance and used a single total PAME score per site.

Five of the nine studies found a positive relationship between PAME scores and biodiversity outcomes; the other four, including the three studies that measured impact using a counterfactual comparison, found no correlation [37,41,42]. It remains unclear whether this lack of correlation with the impact of PAs is real, meaning either that PA management has no impact on biodiversity outcomes, or more plausibly that good management, as measured by PAME scores, is necessary but not sufficient to ensure effective conservation [37]. Alternatively, the lack of a relationship may be owing to limitations related to small sample sizes, PAME data characteristics and assessment process, or the manner in which PAME data were used in the analysis.

## 6. Overcoming constraints on the use of protected area management effectiveness data in impact assessment

Previous studies using PAME data for impact assessment have suffered from small sample sizes. However, management data are now collated and available for over 9000 PAs, predominantly in biodiverse tropical regions, potentially allowing for more extensive analysis.

PAME methodologies are a useful management tool for PA managers with a limited budget. However, they are unlikely to fulfil the data requirements of robust impact evaluation. Methodologies such as the METT and RAPPAM provide a rapid and cost-effective means of communicating the effectiveness of current management and changes in management practices over time to managers and stakeholders. The approach can be easily implemented across a wide range of PA types. However, PAME tools use a relatively simple subjective, ordinal scoring system with limited collection of validating information or systematic auditing of results.

Furthermore, owing to the self-assessment nature of PAME methodologies, the process used to gather expert knowledge for PAME assessment has significant influences on the results and their credibility [45]. Especially where funding for PAME assessments is not ring-fenced within project budgets, PAME assessments may be conducted rapidly with the minimum number of participants, reducing their robustness. The use of PAME methodologies as performance indicators by some conservation donors may encourage funding recipients to deliver overly positive self-assessments at the end of a project. To improve the credibility of PAME scores, we suggest that standardized, robust operating guidelines need to be developed and applied [45,46], including on the selection and training of assessors (ensuring a range of expertise and views and standardized interpretation of indicators), on the format of the assessment procedure (allowing for free debate), and on the duration of the assessment (allowing for thorough deliberation and peer review). Where assessments are conducted as part of donor funding requirements, donors could insist on procedural standards being met and provide specific funding for PAME assessments within project budgets.

Most previous analyses of PAME results in relation to outcomes and impacts have calculated a total PAME score for each PA (table 2), and used this total score as an indicator of management performance. This provides a relatively crude measure of effectiveness that may hide crucial differences in aspects of management; for example, a PA with high scores for budget and staffing questions but low scores for PA outreach and tourism questions would have the same overall score as another PA where the opposite is true. We would advise the use of aggregated and/or weighted scores for different elements of management (such as planning, inputs, process and outputs), and excluding social and biological outcome scores where independent, reliable, empirical data on outcomes and (preferably) impact are available.

Current studies have generally relied upon one-off assessments using PAME data and biodiversity outcome measures from the same period. Static data on PA management may hide important improvements or declines in management over the time period being studied. In addition, the effects of management interventions on outcomes of PAs will rarely be instantaneous; for example, increases in hunted mammal populations may only be measurable years after a reduction in poaching has been achieved. Over 3600 PAs globally now have repeat assessments held in the GD-PAME, of which over 2100 PAs have been assessed three or more times (although over 30% of these are from Australia). Therefore, the impact of changes in management on biodiversity outcomes could now be considered. Continued collection of PAME data through repeat assessments from the same PAs, leading up to and continuing after 2020, will be critical to assemble a significant global time-series dataset.

## 7. Potential use of protected area management effectiveness for measuring and understanding protected area impacts

The large amount of data now held in the GD-PAME provides an opportunity to measure the impact of PA management on biodiversity outcomes at the global scale. Suitable large-scale datasets on biodiversity outcomes, such as changes in terrestrial and marine species populations (e.g. living planet index (LPI), [47]; The Sea Around Us [48] and Global Forest Change [49]), as well as remote-sensed measures of change in human pressure, potentially allow the impacts of PAs to be measured at a global scale, using quasi-experimental methods to construct appropriate counterfactuals [50,51]. However, these approaches consider only the effect of PA presence and suggest that, whereas PAs are effective, effect sizes are often small. Potentially, PAME data enable a further step, allowing impact of improvements in PA management to be investigated. However, the use of global biodiversity data to assess PA impacts only permits the assessment of outcomes for the values represented by the data—these may not reflect the main objectives for PAs.

Studies of the effects of PA management at the local or system level can investigate whether specific management interventions achieve desired management goals; studies could focus, for instance, on the effect of a weed-eradication programme, or law-enforcement efforts to reduce tiger poaching. For rigorous impact evaluation, we would require time-series data on the management interventions, together with time-series data on expected outcomes (i.e. density of weeds, tiger numbers) from both within the PA and an appropriate counterfactual. Some PAME methodologies such as SOP do collect data on individual management interventions such as pest control, as well as data on changes in specific outcomes such as pest population estimates, but do not collect data from appropriate counterfactuals. Data on counterfactuals are unlikely to exist for many PAs, owing to budget, time and staff constraints, and because counterfactual thinking is not mainstream within the conservation community [16]. Measurement of the impacts of PAs relative to appropriate counterfactuals may also be unnecessary for management purposes in some cases. For example, strict law enforcement has been critical in the recovery of tigers in Indian PAs since the 1970s, but there is limited potential for counterfactual analysis because, in many areas, tigers are found only in PAs [52,53].

Even when quasi-experimental methods cannot be used to assess impacts in comparison with appropriately selected counterfactuals, instilling counterfactual thinking into adaptive management processes is a useful way for park managers and advisors to be more explicit about the assumptions they are making, and the possible impacts that are caused by specific interventions. Some PAME evaluations such as SOP are already designed to foster counterfactual thinking, by asking PA managers whether they consider changes in outcomes to be owing to management interventions or external influences. In comparison, tools such as METT and RAPPAM that are relatively weak at capturing outcome-level data (table 1) may benefit from additional questions on outcomes and their causes.

## 8. Conclusion

The establishment and management of PAs remain a primary strategy for biodiversity conservation involving the investment of substantial effort and resources by a wide range of institutions and stakeholders [54]. Over the past two decades, attention has increasingly focused on assessing the effectiveness of management in these sites [12,14], especially to support adaptive improvements to management. The substantial body of assessment results from these ongoing efforts has been consolidated in the GD-PAME, which contains records from around 10% of the world's PAs, with higher proportions within the biodiverse tropics. This database potentially provides an important dataset for investigating the overall effectiveness of PAs, including the potential impacts of PA management interventions on biodiversity outcomes.

Different monitoring approaches and tools will meet different needs; there is no one monitoring and evaluation approach that fits all conservation efforts [55]. The majority of PAME methodologies have been designed to provide a rapid assessment tool for adaptive management. PA managers and other users of PAME assessments at the site and system levels generally report that the assessment process and the findings are useful [25,56]. PAME assessment is a valuable management tool where the process is robustly implemented [57], and information is interpreted within the context of local decision-making [58]. PAME has also been used as a way to instil a ‘learning culture’ within park management agencies [59], and can further lead to the development of nationally applicable standards, allowing greater national ownership [36]. In addition, PAME data are, and will continue to be, an important dataset for reporting on progress towards the management effectiveness element of Aichi Target 11.

Although not their primary purpose, PAME data have been used for both performance and impact evaluation (table 3). However, our review suggests that PAME methodologies may be of limited application in this regard. The subjective and ordinal nature of the GD-PAME data, combined with the paucity of data from appropriate counterfactuals, means that the PAME data are not ideally suited to the needs of scientific impact assessment. Unfortunately, given the reality of limited PA budgets, capacity and staffing [60], it is unlikely that, in the absence of stronger policy or other incentives, PA authorities will prioritize collecting data for scientific impact evaluation.

**Table 3.** Five principal evaluation types, after Mascia *et al.* [15], and the use of PAME data to answer the principal question addressed by each type.

evaluation type (after Mascia [15])	ambient monitoring	management assessment	performance evaluation	impact evaluation	systematic review
principal type of question addressed	What is the state of ambient social and/or environmental conditions, and how are these conditions changing over time and space?	What are the management inputs, activities, and outputs associated with a conservation intervention, and how are these changing over time?	To what extent is a conservation intervention making progress toward its specified objectives for activities, outputs, and outcomes?	What intended and unintended impacts are causally induced by a conservation intervention?	What is the state of the evidence for the impacts of a conservation intervention and what does this evidence say about intervention impacts?
use of PAME data	n.a.	PAME methodologies capture management inputs, activities, and outputs; over 3700 PAs have time-series data. Management assessment is one of the original purposes of PAME methodologies	used by conservation donors (such as the GEF) to measure project performance, in terms of increases in PA management scores over time	to investigate correlations between PA management variables and PA impact as measured using independent datasets on biodiversity	independent impact assessments using PAME data can be combined in systematic review
scale	n.a.	used at all scales; for adaptive management at the site or system level, and to measure progress towards global conservation goals	site- or system-level performance, depending on the scope of the project; combined data can be used to measure the global performance of donor funding programmes	potential for global-scale analyses. Site-level quantitative analyses often hampered by lack of counterfactual data, but counterfactual thinking can still be used in site management	multiple PAs, depending on previous studies

Despite the above-noted challenges, we strongly believe there is a continued need for rigorous scientific impact evaluations of PAs and individual management interventions. There is an emerging evidence base that PAs do work, especially when they are well managed [50,61,62], but understanding what constitutes good management is an ongoing challenge. The GD-PAME provides a global database on results associated with key management interventions, including time-series data for an increasing number of sites. When suitably combined with independent measures of PA impact that have employed appropriate counterfactual methodologies, PAME data can help increase our understanding of the impact of aspects of PA management on conservation outcomes.

## Supplementary Material

Supplementary Materials S1 - S7

## Data accessibility

Data used for the analyses presented in this paper can be obtained from Naomi Kingston, Head of Programme, Protected Areas Programme, UNEP-WCMC. E-mail: naomi.kingston@unep-wcmc.org
