# Supplementary material for: Measuring impact of protected area management interventions: current and future use of the Global Database of Protected Area Management Effectiveness
Source: Philos Trans R Soc Lond B Biol Sci. 2015 Nov 5;370(1681):20140281. doi: 10.1098/rstb.2014.0281 (PMC4614737; doi:10.1098/rstb.2014.0281)
Supplement: Supplementary Materials S1 - S7 [file rstb20140281supp1.pdf]

**Measuring impact of protected area management interventions: current and future use of the Global Database of Protected Area Management Effectiveness**

Lauren Coad, Fiona Leverington, Kathryn Knights, Jonas Geldmann, April Eassom, Valerie Kapos, Naomi Kingston, Marcelo de Lima, Camilo Zamora, Ivon Cuardros, Christoph Nolte, Neil D. Burgess, Marc Hockings

*Phil. Trans. R. Soc. B.* doi: 10.1098/rstb.2014.0281

## Supplementary Materials

S1: The GD-PAME database structure in detail
S2: PAME Headline Indicators
S3: The PAME management cycle
S4: Methods for calculating PAME coverage for countries and ecoregions
S5: Brief description of the 15 most frequently applied PAME methodologies
S6: Literature review methodology
S7: Full descriptions of four widely applied methodologies: METT, RAPPAM, EOH, SOP

© The Authors under the terms of the Creative Commons Attribution License http://creativecommons.org/licenses/by/3.0/, which permits unrestricted use, provided the original author and source are credited.

## S1: GD-PAME database structure in detail

The content of the GD-PAME can be viewed as nested 'levels' of groupings, beginning with methodologies as the highest level, moving down through studies to the lowest level, which is each individual assessment (Fig S1). Methodologies have been recorded with details of their origin, purpose and characteristics. Where methodologies have been applied in the field, one or more studies are recorded (a one-to-many methodologies to study link), and every assessment is allocated to a study (a one-to-many studies to assessment link). A study represents a set of related assessments that are part of a specific evaluation project (such as a state of parks report or an international study of world heritage sites) or applications of a methodology over time in one country.

Indicator information is linked to the methodology. Each individual indicator is entered, along with contextual information about where it sits in the survey tool. The type of scoring and the scale is also entered. Each indicator is allocated according to one headline indicator in the common reporting format. This information is critical to enable translation of raw data into the common reporting format, to allow for analysis across the different methodologies. Indicators are also coded according to elements of the WCPA framework and to the dimensions of management. This allows us to quickly analyse which elements, themes and headline indicators are in different methodologies, and to produce reports that analyse the data under different heading according to need.

The metadata for each assessment (see Fig S1) is recorded separately to the results, with the metadata linking to a results record in cases where the raw results data have been provided. Raw results from the different methodologies are translated into the set of common indicators, so that the results for all methodologies are in the common reporting format. The metadata contains details of the PA to which the assessment applies. The assessment unit is usually an individual PA, but in some cases an overlying PA designation such as World Heritage Area may include multiple protected areas such as national parks or wildlife reserves. PAs are also sometimes assessed in groups as management units that do not reflect an overlying designation, e.g. at a 'landscape' scale. In addition, Important Bird Areas (IBA) assessed by Birdlife International are included in the GD_PAME as one assessment unit, provided they overlap by 75% or more with PAs. Each assessment unit –a PA, group of PAs or an IBA – is given a unique ID code, allowing the identification of repeat assessments or the use of multiple methodologies to assess the same unit.

The database is linked to the WDPA using the WDPA ID code. Each assessment links to one or more records on the WDPA codes table, which contains a record for each WDPA ID code that represents an area that is contained in the assessed unit. Many assessments are for PAs that have not yet been recorded in the WDPA, in which case a 'no data' marker is used in the WDPA codes table.

**Figure S1: GD-PAME database structure and key variables.**

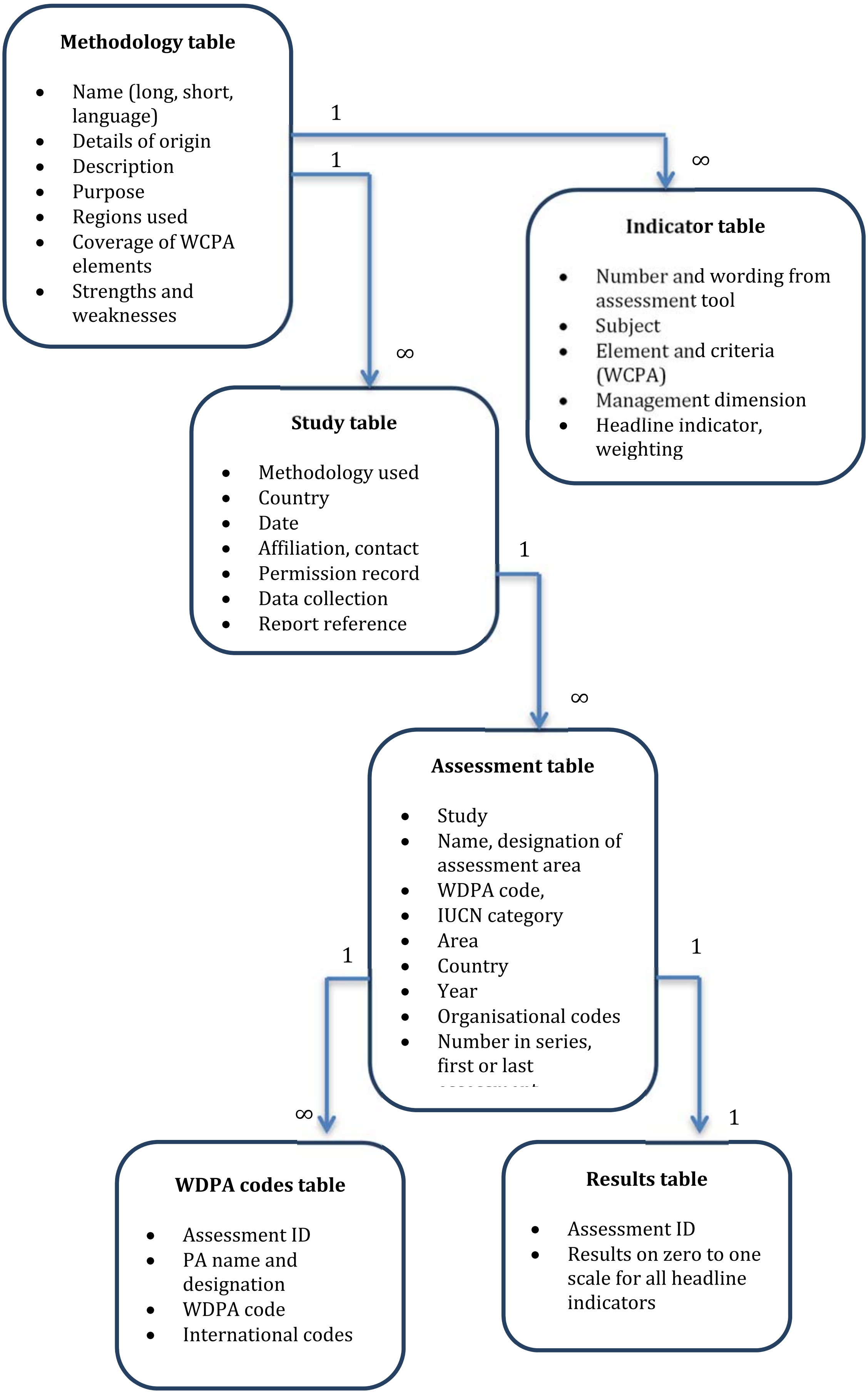

**S2: PAME headline indicators**

| ID | Headline indicators | element |
|---|---|---|
| 1 | Level of significance | context |
| 2 | Five important values | context |
| 3 | Level of extent and severity of threats | context |
| 4 | Trend of threats | context |
| 5 | Five important threats | context |
| 6 | Constraint or support by external political and civil environment | context |
| 7 | Main constraining factors of external political and civil environment | context |
| 8 | Park gazettal | planning |
| 9 | Marking and security/ fencing of park boundaries | planning |
| 10 | Appropriateness of design | planning |
| 11 | Management plan | planning |
| 12 | Adequacy of staff numbers | input |
| 13 | Adequacy of current funding | input |
| 14 | Security/ reliability of funding | input |
| 15 | Adequacy of infrastructure, equipment and facilities | input |
| 16 | Adequacy of relevant and available information for management | input |
| 17 | Effectiveness of governance and leadership | process |
| 18 | Model of governance | process |
| 19 | Effectiveness of administration including financial management | process |
| 20 | Management effectiveness evaluation undertaken | process |
| 21 | Adequacy of building and maintenance systems | process |
| 22 | Adequacy of staff training | process |
| 23 | Staff/ other management partners skill level | process |
| 24 | Adequacy of hr policies and procedures | process |
| 25 | Staff morale | process |
| 26 | Adequacy of law enforcement capacity | process |
| 27 | List (up to) five main issues for law enforcement | process |
| 28 | Involvement of communities and stakeholders | process |
| 29 | Communication program | process |
| 30 | Appropriate program of community benefit/ assistance | process |
| 31 | List community benefit/ assistance program | process |
| 32 | Sustainable resource use - management and audit | process |
| 33 | Visitors catered for and impacts managed appropriately | process |
| 34 | Character of visitor facilities and services | process |
| 35 | Level of visitor use | context |
| 36 | Natural resource and cultural protection activities undertaken | process |
| 37 | Research and monitoring of natural/ cultural management | process |
| 38 | Achievement of set work program | output |
| 39 | Results and outputs have been produced | output |
| 40 | Proportion of stated objectives achieved | outcome |
| 41 | Conservation of nominated values -condition | outcome |
| 42 | Conservation of nominated values - trend | outcome |
| 43 | Effect of park management on local community | outcome |
| 44 | None applicable | 0 |
| 45 | Threat monitoring | process |
| 46 | Adequacy of p.a. legislation and other legal controls | planning |
| 47 | Tenure security | planning |

**S3: Steps around a management cycle, depicting the 6 main PAME elements**

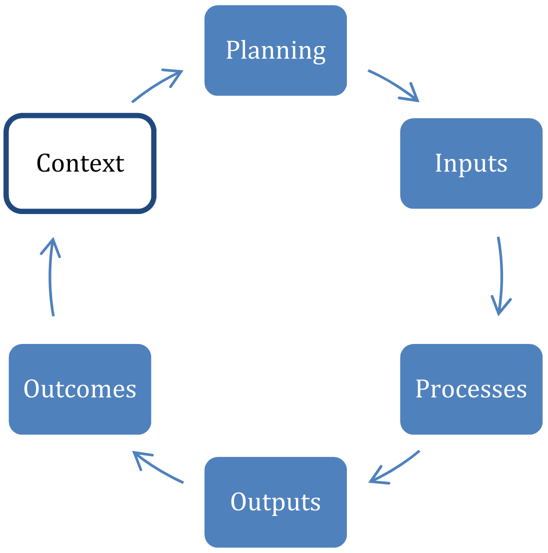

**S4: Detailed Methods for calculation of PAME coverage**

**S4A: Methods for calculations of PAME coverage of protected areas by country**

The following text outlines the methods used to calculate PAME coverage per country using the World Database of Protected Areas and the GD-PAME database

1. Remove unwanted assessments:
   a. Mexican assessments falling outside of the protected area network labeled 'EXCLUDE METT non-PA'.
   b. The following studies: Biosphere Reserve Stockholm, Birdlife batch 1, Birdlife batch 2 and GOBI Survey.
2. Where possible assign missing WDPA codes.
3. Within Microsoft Access group the PAME assessment data by WDPAID code in order to remove duplicate records and export the resulting table as a .dbf. Table S3A provides basic statistics on the data included within this analysis.

**Table S4A** – Basic statistics on the number of assessments included with the analysis

| | |
|---|---|
| Number of PAME Assessments | 15672 |
| Number of unique WDPA ID's | 5456 |
| Number of records without WDPA ID for which area is reported | 866 |
| Number of records without WDPA ID or area information i.e. records that could not be included within the analysis | 1790 |

4. Upload the latest WDPA polygon and point shapefiles to ArcGIS.
5. Run a definition query on both the point and polygon shapefiles to select out only those records with the status 'Designated'.

**Assessed Area**

*i) Polygon and Point Data*

6. Within ArcGIS join the above mentioned PAME assessment .dbf file to both the WDPA point and polygon shapefiles separately using the attribute WDPAID (ensure the keep only matching records option is ticked).
7. Buffer each point within the 'assessed' point dataset using radius values calculated from the 'REP_AREA' field (Equation: sqt('REP_AREA'/$\pi$)). In order to avoid distortion perform the buffer in a geographic coordinate system e.g. WGS84
8. Merge the buffered point dataset with the 'assessed' polygon shapefile to create a single, new output dataset containing all 'assessed' WDPA information. Combine ISO3 information from both the point and polygon dataset into a single field entitled 'Country'.
9. Dissolve the above dataset by ISO3 thus removing any overlaps.
10. Add an additional field to the attribute table entitled 'Area' selecting the type double.
11. Populate this field using the calculate geometry tool. Set the property to 'Area' and the units to $km^2$. Before performing this calculation ensure that the dataset is in the projected co-ordinate system Molleweide.
12. Export the area values

NOTE: Countries containing a large number of protected areas may have multiple records after the initial dissolve. Add together the area values for records with the same ISO3 code.

*ii) Transboundary Data*

13. Following on from step 9 export all transboundary protected areas (i.e. areas with more than one associated ISO3 code) into a new shapefile.
14. Perform an intersect between the transboundary protected area shapefile and either a country boundary dataset for terrestrial PA's or an EEZ boundary dataset for marine PA's in order to determine what proportion of the PA lies within each country.
15. Add an additional field to the attribute table entitled 'Area' selecting the type double.

16. Populate this field using the calculate geometry tool. Set the property to 'Area' and the units to $km^2$.
17. Export the area values

*iii) Records with no WDPA_ID*

18. For those records without a WDPA_ID use the area reported in the PAME assessment (if provided), remembering to convert the area from hectares to $km^2$.
19. Sum these area values per ISO3 code.

*iv) Final Calculation*

20. Add together the area values calculated in sections i), ii) and iii) for records with matching ISO3 codes in order to determine the total area assessed within protected areas per country.

**Total Area**

*i) Polygon and Point Data*

21. Following on from step 5 - Buffer each point within the WDPA point dataset using radius values calculated from the 'REP_AREA' field (Equation: sqt('REP_AREA'/π)). In order to avoid distortion perform the buffer in a geographic coordinate system e.g. WGS84.
22. Merge the buffered WDPA point dataset with the WDPA polygon shapefile to create a single, new output dataset containing all WDPA information. Combine ISO3 information from both the point and polygon dataset into a single field entitled 'Country'.
23. Dissolve the above dataset by ISO3 thus removing any overlaps.
24. Add an additional field to the attribute table entitled 'Area' selecting the type double.
25. Populate this field using the calculate geometry tool. Set the property to 'Area' and the units to $km^2$. Before performing this calculation ensure that the dataset is in the projected co-ordinate system Molleweide.
26. Export the area values

NOTE: Countries containing a large number of protected areas may have multiple records after the initial dissolve. Add together the area values for records with the same ISO3 code.

*ii) Transboundary Data*

27. Following on from step 23 - export all transboundary protected areas (i.e. areas with more than one associated ISO3 code) into a new shapefile.
28. Perform an intersect between the transboundary protected area and either the country boundaries for terrestrial PA's or the EEZ boundaries for marine PA's in order to determine what proportion of the PA lies within each country.
29. Add an additional field to the attribute table entitled 'Area' selecting the type double.
30. Populate this field using the calculate geometry tool. Set the property to 'Area' and the units to $km^2$.
31. Export the area values

*iv) Final Calculation*

32. Add together the area values calculated in sections i) and ii) for all records with matching ISO3 codes and add to this the area values calculated in step 19 (area of assessed records with no WDPAID). You now have the total area of PA's per country.

**Percentage Area Assessed**

33. In order to determine the percentage area of protected land assessed within each country, divide the total area assessed (step 20) by the total area of protected land (output 32) for each ISO3 code separately and then times the answer by 100.

    (Total assessed area (per ISO) \ Total PA area (per ISO)) * 100
34. Categorise the data into the following classes: 0, <10%, 10-30%, 30-60% and >60%
35. Save the results as .dbf
36. Within ArcGIS join the above results .dbf file to a country boundary shapefile using the attribute ISO3.
37. Select a suitable symbology to display the results

**Table S4B: Tabulated data per country on PAME assessments per country**

| Country | % Area Assessed | Country | % Area Assessed | Country | % Area Assessed | Country | % Area Assessed |
|---|---|---|---|---|---|---|---|
| ABNJ | 0.0 | CCK | 0.0 | GIB | 0.0 | LBY | 0.0 |
| ABW | 0.0 | CHE | 21.6 | GIN | 22.9 | LCA | 17.6 |
| AFG | 0.0 | CHL | 12.4 | GLP | 0.0 | LIE | 0.0 |
| AGO | 37.0 | CHN | 27.8 | GMB | 99.5 | LKA | 3.9 |
| AIA | 0.0 | CIV | 25.5 | GNB | 37.3 | LSO | 98.3 |
| ALA | 0.0 | CMR | 78.6 | GNQ | 58.4 | LTU | 11.5 |
| ALB | 17.7 | COD | 53.1 | GRC | 1.5 | LUX | 33.7 |
| AND | 0.0 | COG | 31.4 | GRD | 58.4 | LVA | 30.7 |
| ANT | 0.0 | COK | 0.0 | GRL | 0.0 | MAF | 0.0 |
| ARE | 0.0 | COL | 58.0 | GTM | 83.0 | MAR | 1.1 |
| ARG | 31.6 | COM | 0.0 | GUF | 45.0 | MCO | 0.0 |
| ARM | 32.9 | CPV | 99.2 | GUM | 0.0 | MDA | 69.5 |
| ASM | 0.0 | CRI | 79.3 | GUY | 20.3 | MDG | 51.8 |
| ATA | 0.0 | CUB | 47.9 | HKG | 0.0 | MDV | 0.0 |
| ATF | 0.0 | CUW | 0.0 | HND | 65.6 | MEX | 59.0 |
| ATG | 0.0 | CXR | 0.0 | HRV | 17.9 | MHL | 0.0 |
| AUS | 15.1 | CYM | 0.0 | HTI | 93.7 | MKD | 96.3 |
| AUT | 2.7 | CYP | 0.0 | HUN | 3.4 | MLI | 93.3 |
| AZE | 30.8 | CZE | 19.9 | IDN | 40.0 | MLT | 0.0 |
| BDI | 51.6 | DEU | 16.4 | IND | 20.8 | MMR | 15.9 |
| BEL | 0.6 | DJI | 1.4 | IOT | 0.0 | MNE | 39.6 |
| BEN | 81.5 | DMA | 4.6 | IRL | 0.0 | MNG | 77.6 |
| BES | 0.0 | DNK | 16.5 | IRN | 4.5 | MNP | 0.0 |
| BFA | 76.2 | DOM | 57.6 | IRQ | 0.0 | MOZ | 25.5 |
| BGD | 21.9 | DZA | 82.9 | ISL | 0.2 | MRT | 94.3 |
| BGR | 10.8 | ECU | 84.6 | ISR | 0.0 | MSR | 0.0 |
| BHR | 0.0 | EGY | 56.9 | ITA | 4.1 | MTQ | 0.0 |
| BHS | 99.4 | ERI | 0.0 | JAM | 89.0 | MUS | 71.5 |
| BIH | 53.7 | ESH | 0.0 | JEY | 0.0 | MWI | 57.8 |
| BLM | 0.0 | ESP | 5.1 | JOR | 91.4 | MYS | 26.9 |
| BLR | 29.0 | EST | 7.8 | JPN | 1.2 | MYT | 0.0 |
| BLZ | 86.4 | ETH | 13.9 | KAZ | 52.4 | NAM | 42.4 |
| BMU | 0.0 | FIN | 68.3 | KEN | 12.1 | NCL | 0.0 |
| BOL | 56.3 | FJI | 2.1 | KGZ | 4.9 | NER | 51.9 |
| BRA | 33.5 | FLK | 0.0 | KHM | 55.4 | NGA | 5.9 |
| BRB | 0.0 | FRA | 10.2 | KIR | 99.8 | NIC | 48.9 |
| BRN | 0.0 | FRO | 0.0 | KNA | 0.0 | NIU | 50.8 |
| BTN | 53.7 | FSM | 0.0 | KOR | 62.0 | NLD | 15.4 |

| Country | % Area Assessed | Country | % Area Assessed | Country | % Area Assessed | Country | % Area Assessed |
|---|---|---|---|---|---|---|---|
| BVT | 0.0 | GAB | 57.6 | KWT | 0.0 | NOR | 2.1 |
| BWA | 34.6 | GBR | 7.3 | LAO | 86.7 | NPL | 67.9 |
| CAF | 68.6 | GEO | 85.0 | LBN | 0.6 | NZL | 3.0 |
| CAN | 13.0 | GHA | 15.0 | LBR | 94.9 | OMN | 0.0 |
| PAK | 16.7 | SDN | 0.0 | SWZ | 0.0 | UGA | 14.7 |
| PAN | 95.0 | SEN | 29.1 | SYC | 86.0 | UKR | 26.8 |
| PER | 34.8 | SGP | 3.7 | SYR | 42.3 | UMI | 0.0 |
| PHL | 13.1 | SGS | 0.0 | TCA | 0.0 | URY | 19.2 |
| PLW | 63.3 | SHN | 0.0 | TCD | 61.9 | USA | 18.8 |
| PNG | 97.8 | SJM | 0.0 | TGO | 29.1 | UZB | 15.0 |
| POL | 1.7 | SLB | 67.3 | THA | 20.4 | VCT | 57.8 |
| PRI | 0.0 | SLE | 19.4 | TJK | 81.7 | VEN | 34.5 |
| PRK | 3.3 | SLV | 18.7 | TKL | 0.0 | VGB | 0.0 |
| PRT | 0.2 | SOM | 0.0 | TKM | 62.5 | VIR | 17.7 |
| PRY | 10.7 | SPM | 0.0 | TLS | 0.0 | VNM | 22.9 |
| PYF | 0.0 | SRB | 64.7 | TON | 0.0 | VUT | 38.3 |
| QAT | 0.0 | SSD | 60.3 | TTO | 0.0 | WLF | 0.0 |
| REU | 0.0 | STP | 0.0 | TUN | 16.1 | WSM | 59.7 |
| ROU | 37.1 | SUR | 98.0 | TUR | 86.9 | YEM | 14.8 |
| RUS | 42.0 | SVK | 14.0 | TUV | 0.0 | ZAF | 24.9 |
| RWA | 43.6 | SVN | 14.5 | TWN | 0.0 | ZMB | 32.1 |
| SAU | 0.0 | SWE | 13.5 | TZA | 39.9 | ZWE | 20.5 |

**S4C: Methods for calculation of PAME Assessment coverage per ecoregion**
**Total Area**

Total protected area ecoregion coverage values were calculated by JRC for the 2014 Protected Planet Report (UNEP WCMC 2014). These values were calculated as follows:

1. Start with the WDPA updated version to release in August 2014.
2. Append restricted polygons and points to the polygon and file points respectively. These are sites from countries that do not allow public download or commercial use of their data but who do allow use for UNEP-WCMC analyses.
3. Check and correct geometry errors using ArcGIS tools or similar tools.
4. Remove all points and polygons with STATUS = “not reported” and STATUS = “proposed”
5. Remove all UNESCO MAB Biosphere reserves. This is because they may include large buffer areas that do not meet the definition of protected areas and they largely overlap with existing protected areas.
6. Remove points with no reported area. It is not possible to calculate area for protected areas without polygon and reported area.
7. Create buffers around points.
    i. Add two new fields in the point data set’s attribute table called ‘Radius’ (field type: double) and ‘Buffer_Field’ (field type: string).
    ii. Use field calculator to calculate the radius of the points’ ‘Reported Area’ into the new field ‘Radius’. The expression used in ArcGIS 10.2 with Python scripting is math.sqrt( !REP_AREA! / math.pi)

iii. Use field calculator to copy the values from the radius field into the new field ‘Buffer_Field’ and add the string ‘kilometers’ so that you have both the radius and the word kilometres in the field. These should be separated by a space. The expression looks like: str(!Radius! + “ kilometres” using Python script
iv. Use the Buffer Analysis Tool in the Geoprocesseing Toolkit to buffer the points. The field used to create the buffers will be ‘Buffer_Field’.
v. Create a new field called ‘Buffer_Area’ (field type: double) to check the accuracy of the Buffers. **Note** that the projection used to calculate the geometry will affect the validity of the area calculation. The standard Molleweide projection decreases in accuracy with the size of the Reported Area, with the largest differences up to 1.5% of the Reported Area. The most accurate global projection is the Molleweide Auxillary Sphere set to Auxillary Sphere Type 3 (has to be customised in ESRI). The greatest difference here was 0.00014% of the Reported Area.

8. Merge buffered points to polygons to create one WDPA polygon layer and check and repair any geometry issues.
9. Use the “Dissolve” function to get one “flat” WDPA layer for analyses.
10. WDPA layer ready for coverage analyses.
11. Intersect WDPA layer with the WWF ‘Ecoregions of the World’ datalayer
12. Add an additional field to the attribute table entitled ‘Area’ selecting the type double.
13. Populate this field using the calculate geometry tool. Set the property to ‘Area’ and the units to $km^2$. Before performing this calculation ensure that the dataset is in the projected co-ordinate system Molleweide.
14. Export the total area values.

**Assessed Area**

15. Remove unwanted assessments from the PAME database:
    a. Mexican assessments falling outside of the protected area network labelled ‘EXCLUDE METT non-PA’.
    b. The following studies: Biosphere Reserve Stockholm, Birdlife batch 1, Birdlife batch 2 and GOBI Survey
16. Where possible assign missing WDPA codes.
17. Within Microsoft Access group the PAME assessment data by WDPAID code in order to remove duplicate records and export the resulting table as a .dbf.
18. Within ArcGIS join the above mentioned PAME assessment .dbf file to the merged buffered points to polygons WDPA data layer created in step 8 (ensure the keep only matching records option is ticked).
19. Use the “Dissolve” function to get one “flat” WDPA layer for analyses.
20. Intersect WDPA layer with the WWF ‘Ecoregions of the World’ datalayer
21. Add an additional field to the attribute table entitled ‘Area’ selecting the type double.
22. Populate this field using the calculate geometry tool. Set the property to ‘Area’ and the units to $km^2$. Before performing this calculation ensure that the dataset is in the projected co-ordinate system Molleweide.
23. Export the assessed area values.

**Percentage Area Assessed Calculation**

1. In order to determine the percentage area of protected land assessed within each ecoregion, divide the total area assessed (step 23) by the total area of protected land (output 14) for each ecoregion separately and then times the answer by 100.
   (Total assessed area (per ecoregion) \ Total PA area (per ecoregion)) * 100
2. Categorise the data into the following classes: <10%, 10-30%, 30-60% and >60%
3. Save the results as .dbf

4. Within ArcGIS join the above results .dbf file to a country boundary shapefile using the attribute ECO ID.
5. Select a suitable symbology to display the results

UNEP-WCMC (2014). Global statistics from the World Database on Protected Areas (WDPA), August 2014.Cambridge, UK: UNEP- WCMC.

**Table S4D: Percentage of PAs PAME assessed per terrestrial biome**

| Terrestrial biome name | Total PA area ($km^2$) | Assessed PA area ($km^2$) | % Area Assessed |
|---|---|---|---|
| Boreal Forests/ Taiga | 1570568.62 | 347116.82 | 22 |
| Deserts and Xeric Shrublands | 3382966.68 | 799696.19 | 24 |
| Flooded Grasslands and Savannas | 339170.15 | 131295.16 | 39 |
| Mangroves | 97982.61 | 49833.64 | 51 |
| Mediterranean Forests, Woodlands and Scrub | 512190.25 | 57319.29 | 11 |
| Montane Grasslands and Shrubland | 1393007.36 | 539478.41 | 39 |
| Temperate Broadleaf and Mixed Forest | 1540765.66 | 301369.34 | 20 |
| Temperate Coniferous Forest | 687694.43 | 168073.39 | 24 |
| Temporate Grasslands, Savannas and Shrubland | 456516.79 | 80551.16 | 18 |
| Tropical and Subtropical Coniferous Forest | 83513.25 | 35406.88 | 42 |
| Tropical and Subtropical Dry Broadleaf Forest | 290896.21 | 117590.81 | 40 |
| Tropical and Subtropical Grasslands, Savannas and Shrubland | 2973704.33 | 1184134.61 | 40 |
| Tropical and Subtropical Moist Broadleaf Forest | 4712330.83 | 2122586.32 | 45 |
| Tundra (excluding antarctic) | 1812734.35 | 271031.34 | 15 |

**S5: Brief description of the 15 most frequently applied PAME methodologies**

| Full name | Category * | Number of protected areas assessed | Number of countries assessed | Description/ notes |
|---|---|---|---|---|
| Management Effectiveness Tracking Tool (METT) (1) | 1,2,3 | 3834 | 124 | Scorecard to track progress of individual protected areas over time. |
| Birdlife Important Bird Area Monitoring (2) | 1 | 2998 | 137 | Primarily developed to track and respond to threats, understand the status and trends of biodiversity, and assess the effectiveness of conservation efforts. Contains a small suite of management effectiveness indicators but was not specifically designed for this purpose. |
| Rapid Assessment and Prioritisation of Protected Area Management (RAPPAM) (3) | 1,3 | 1915 | 65 | Designed for broad-level comparisons among many protected areas which together make a protected areas network or system. Applied by NGOs and/or national governments, for example by Brazil. Numerous reports available. |
| Australian SOP and related (4) | 3 | 1533 | 1 | Methodologies from four agencies - NSW, VIC, Qld, NT – repeated up to four times. |
| World Heritage Areas Outlook Report (5) | 2 | 228 | 95 | Desktop review using expert knowledge of natural World Heritage sites, based on IUCN WCPA Management Effectiveness Evaluation Framework. |
| Global Biosphere Reserve Survey(6) | 4 | 225 | 74 | Academic survey of over 200 biosphere reserves in 70 countries. No repeat assessments. |
| PROARCA/CAPAS scorecard evaluation (7) | 2,3 | 209 | 6 | Scorecard methodology supported and led by TNC with USAID funding, but high level of involvement of agencies in Central America. Conducted in six countries with up to six repeat assessments. |
| Parks in Peril (PiP) Site Consolidation Scorecard (8) | 1 | 83 | 17 | PiP fostered the local support necessary for protecting 'paper parks' using a process called site consolidation. |
| European diploma (9) | 2 | 72 | | Awarded areas are of particular European interest for its biological, geological and landscape diversity |

| | | | | |
|---|---|---|---|---|
| Belize Management Effectiveness (10) | 3 | 69 | 1 | Detailed assessments of all protected areas in system on two occasions. |
| German Nature Parks Quality Campaign (11) | 3 | 64 | 1 | Performance assessment questionnaire assessing performance in relation to environmental protection, recreation and tourism, education and communication and sustainable regional development as well as protected area planning and management. |
| AEMAPPS: MEE with Social Participation – Colombia (12) | 1,3 | 53 | 1 | Institutionalised PAME system used by the Sisyema de Parques Nacionales Naturales de Colombia. Initially developed with WWF, it is now regularly applied by the management agency across all national parks in Colombia. |
| USA State of Parks (13), 13a) | 1 | 46 | 1 | Very detailed assessments by NGO of all aspects of park management up to 2011– major purpose of awareness-raising.<br>State of Parks reporting now being undertaken by the National Parks service with online reports available. |
| Korea State of Parks (14) | 3 | 42 | 1 | National system for assessment of PAME in Korean National Parks Service sites, based on an adaptation of State of the Parks assessment systems in Australia. It is not being more widely applied across other protected areas in South Korea. |
| Enhancing our Heritage (15) | 1,2 | 16 | 13 | Very detailed assessments including review of outcomes. Aimed at World Heritage Areas. |

*: 1=NGO assessments, 2=international agencies, 3= national agencies, 4=academic study


1) Stolton, S., Hockings, M., Dudley, N., MacKinnon, K., Whitten, T., et al., 2007. Reporting Progress in Protected Areas A Site-Level Management Effectiveness Tracking Tool: second edition. World Bank/WWF Forest Alliance published by WWF, Gland, Switzerland.
2) BirdLife International, 2006. Monitoring Important Bird Areas: a global framework. Version 1.2. BirdLife International, Cambridge, UK.

3) Ervin, J., 2003. WWF: Rapid Assessment and Prioritization of Protected Area Management (RAPPAM) Methodology. WWF, Gland, Switzerland.
4) Growcock, A., Sutherland, E. and Stathis, P. (2009) ‘Challenges and experiences in implementing a management effectiveness evaluation program in a protected area system’, *Australasian Journal of Environmental Management* 16(4): 218–26.

5) IUCN (2012) Conservation Outlook Assessments - Guidelines for their application to natural World Heritage Sites IUCN. Also see http://www.worldheritageoutlook.iucn.org/methodology
6) Stoll-Kleemann, S. and H. Job (2008). "The Relevance of Effective Protected Areas for Biodiversity Conservation: An Introduction." GAIA - Ecological Perspectives for Science and Society 17(1): 86-89.
7) Corrales, L., 2004. Midiendo el éxito de las acciones en las áreas protegidas de Centroamérica: Medición de la Efectividad de Manejo. PROARCA/APM, Guatemala de la Asunción, Guatemala.
8) The Nature Conservancy Parks in Peril Program, 2004. Measuring success: The Parks in Peril Site Consolidation Scorecard Manual (Updated May 10, 2004).
9) Council of Europe, 2008: http://www.coe.int
10) Young, R., Wolfe, L. and Mc.Farlane, V., 2005. Monitoring Management Effectiveness in Belize's Protected Areas System. Report prepared for the National Protected Areas Policy & System Plan Task Force (NPAPSP). University Research and Evaluation and Galiano Institute for Environmental and Social Research.
11) Köster, U., Wilken, T., Brittner, S., Bausch, T. (2006): 'Nature's Park Quality Campaign', Verband Deutscher Naturparke e.V., Bonn. Available online at www.naturparke.de
12) Medina M, Parques Nacionales Naturales de Colombia y WWF Colombia. (2005) Análisis de Efectividad del Manejo de Áreas Protegidas con Participación Social. AEMAPPS. WWF Colombia, Parques Nacionales Naturales de Colombia, Subdirección Técnica.
13) National Parks Conservation Association State of the Parks Program (2005) Cultural Resources Assessment Methodology. National Parks Conservation Association. http://www.npca.org/about-us/center-for-park-research/stateoftheparks/

National Parks Conservation Association State of the Parks Program (no date) State of the Parks: Natural Resources Assessment and Ratings Methodology. National Parks Conservation Association. http://www.npca.org/about-us/center-for-park-research/stateoftheparks/

13 a) National Parks Service, U.D.o.t.I., 2014. Program brief: State of the Parks Reporting, US Department of the Interior http://www.nps.gov/stateoftheparks/
14) Heo, H.Y., Hockings, M., Shin, W.W., Chung, H.J., Dudley, N., et al., 2010. Management Effectiveness Evaluation of Korea's Protected Area System. Journal of National Park Research 1, 169-179.
15) Hockings, M., Stolton, S., Dudley, N., James, R., Mathur, V., Courrau, J., Makombo, J. and Parrish, J. (2008) Enhancing our Heritage Toolkit: Assessing management effectiveness of natural World Heritage sites. UNESCO World Heritage Papers No. 23. UNESCO, Paris. 104pp.

## S6: Literature review methodology

### Search strategy

The review was conducted following the general guidelines from the Collaboration of Environmental Evidence for systematic reviews [1]. However the search protocol was developed directly for the search and not peer-reviewed.

A PICO (Population, Intervention, Comparator, Outcome) table was constructed to frame the search question (Table S1).

Table S6A. PICO elements of the review question

| Element | Definition |
|---|---|
| Subject population | Spatially references unit of change in amount, coverage, or quality of biodiversity and/or habitat |
| Intervention | Recognized and described Protected Area Management Effectiveness methodology included in the global database on protected area management effectiveness |
| Comparator | Spatial overlap between measures of intervention and subject |
| Outcome | Causal or correlative link between measures of intervention and subject |

### Online databases and catalogues:

Articles were ordered by relevance, where this feature was available, and searches were restricted to papers within the databases' 'conservation' categories to increase the relevance of papers found. Papers accepted based on title were subsequently reviewed by abstract and finally full text.

Thomson Reuthers Web of Knowledge www.webofknowledge.com

### Specialist websites

Library and report sections of the websites were located and reports assessed by title. Potentially relevant sources were downloaded and fully assessed.

- World Wildlife Fund http://wwf.panda.org/about_our_earth/all_publications/
- The Global Environmental Facility https://www.thegef.org/gef/gef_Documents_Publications
- UNESCO http://www.unesco.org/new/en/culture/themes/creativity/creative-cities-network/literature/
- IUCN https://www.iucn.org/about/work/programmes/gpap_home/gpap_capacity2/gpap_litdata/
- NOAA http://www.lib.noaa.gov/researchtools/journals/databases.html
- The Nature conservancy http://www.nature.org

The search was first conducted using ISI Web of Knowledge, and results from specialist websites were subsequently added to the list. This insured that duplicates were removed throughout the search process. Articles that were evaluated to be outside the

scope and question of the systematic review or did not follow the study inclusion criteria or quality assessment were removed from the list, as they were identified.

**Search terms**

Search terms were restricted to only cover the intervention (i.e. "protected areas" and "management effectiveness") to ensure not excluding any studies using an alternative measure of biodiversity.

Table S6B. Search terms used in the review

| Protected Area | Management Effectiveness |
|---|---|
| Community conserved area$ | Management effectiveness |
| Protected area$ | Protected area management effectiveness |
| National park$ | Management effectiveness tracking tool |
| Reserve$ | Rapid Assessment and Prioritization of Protected Areas Management |
| Indigenous* | Parks in peril |
| World Heritage | Enhancing our heritage |
| Important Bird areas | How is your MPA doing |
| Key Biodiversity areas | Conservation action planning |
| | Score card |

**Study inclusion criteria**

Only studies using a PAME methodology included in the GD-PAME were included in the study. We only included studies that assessed a link between the level or change in management effectiveness, as measured using a PAME methodology, and a measure of biodiversity. Only studies in protected areas were included.

**Study characterization & quality assessment**

For all study we evaluated whether there were direct observations of population trends, indices or expert evaluations. All measures were included in the final review. For habitat the remote sensing product or the on-ground evaluation method was assessed.

For all studies we collected information to assess the ability to link input and outcomes and to evaluate their ability to make quantitative or only qualitative evaluations of the effect of protection and secondarily interventions. We recorded characteristics included elements of the following:

- Country and geographical area of study
- Governance factors influencing protection
- Counterfactual scenarios (BACI)
- Contextual factors reported do influence management effectiveness
- Number of species used in the study-design
- Methods for data collection and type of analysis

All of the above was used to critically appraise the included studies and evaluate to what extend results and conclusions were appropriate to support the statements of PA effectiveness as reported in the papers.

Table S6 C. Studies discarded at the level of abstract or full text

| Study | PAME methodology | Outcome / Imapact measure |
|---|---|---|
| Bleher et al. 2006 [2] | No – different management authorities | Yes – rates of illegal logging |
| Bonham et al. 2014 [3] | No – program assessment not using a standard PAME methodology | Yes – deforestation rates |
| Cao et al. 2014 [4] | Yes – adaptation of METT | No – only measures the level of PAME |
| Goodman 2003 [5] | Yes – RAPPAM | No – only measures the level of PAME |
| Izurieta et al. 2011 [6] | No – study of how to apply PAME tools | No – Study of how to apply PAME tools |
| Jacobsen et al. 2008 [7] | Yes – multiple | No – only measures the level of PAME |
| Kolahi et al. 2013[8] | Yes – METT | No – only measures the level of PAME |
| Lu et al. 2012 [9] | Yes – RAPPAM | No – only measures the level of PAME |
| Maypa et al. 2012 [10] | Yes – World bank score card | No – only measures the level of PAME |
| McClanahan et al. 2006 [11] | No – different management authorities | Yes – difference in biodiversity |
| Moore and Walker 2008 [12] | No – but questionnaire was designed based on IUCN management effectiveness guidelines | No – measured the impact of visitors as their observed and perceived behavior |
| Muthiga 2009 [13] | No – different management authorities | Yes – fish biomass |
| Ohnesorge et al. 2013 [14] | No – different types of PA designation | Yes landcover assessments |
| Parr et al. 2008 [15] | No – difference in specific interventions (fire prevention and control of alien species) | No – not sufficient data available |
| Pressey et al. 2002 [16] | No – conservation planning study | No – species richness of areas not related to management |
| Quan et al. 2011 [17] | Yes - METT | No – only measures the level of PAME |

## References

[1] Centre for Evidence-Based Conservation. 2010 Guidelines for Systematic Review in Environmental Management. Version 4.0. Bangor University, UK, Centre for Evidence-Based Conservation; 71 pp. p.

[2] Bleher, B., Uster, D. & Bergsdorf, T. 2006 Assessment of threat status and management effectiveness in Kakamega Forest, Kenya. Biodivers. Conserv. 15, 1159-1177.

[3] Bonham, C., Steininger, M.K., McGreevey, M., Stone, C., Wright, T. & Cano, C. 2014 Conservation Trust Funds, Protected Area Management Effectiveness, and Conservation Outcomes: Lessons from the Global Conservation Fund. Parks 20, 89-100.

[4] Cao, H., Tang, M., Deng, H. & Dong, R. 2013 Analysis of management effectiveness of natural reserves in Yunnan Province, China. International Journal of Sustainable Development & World Ecology 21, 77-84. (doi:10.1080/13504509.2013.786764).

[5] Goodman, P.S. 2003 Assessing management effectiveness and setting priorities in protected areas in KwaZulu-Natal. Bioscience 53, 843-850.

[6] Izurieta, A., Sithole, B., Stacey, N., Hunter-Xenie, H., Campbell, B., Donohoe, P., Brown, J. & Wilson, L. 2011 Developing Indicators for Monitoring and Evaluating Joint Management Effectiveness in Protected Areas in the Northern Territory, Australia. Ecol. Soc. 16. (doi:10.5751/es-04274-160309).

[7] Jacobson, C., Carter, R.W. & Hockings, M. 2008 The status of protected area management evaluation in Australia and implications for its future. Australasian Journal of Environmental Management 15, 202-210.

[8] Kolahi, M., Sakai, T., Moriya, K., Makhdoum, M. & Koyama, L. 2013 Assessment of the Effectiveness of Protected Areas Management in Iran: Case Study in Khojir National Park. Environ. Manage. 52, 514-530. (doi:10.1007/s00267-013-0061-5).

[9] Lu, D.J., Kao, C.W. & Chao, C.L. 2012 Evaluating the Management Effectiveness of Five Protected Areas in Taiwan Using WWF's RAPPAM. Environ. Manage. 50, 272-282. (doi:10.1007/s00267-012-9875-9).

[10] Maypa, A.P., White, A.T., Canares, E., Martinez, R., Eisma-Osorio, R.L., Alino, P. & Apistar, D. 2012 Marine Protected Area Management Effectiveness: Progress and Lessons in the Philippines. Coast. Manage. 40, 510-524. (doi:10.1080/08920753.2012.709465).

[11] McClanahan, T.R., Verheij, E. & Maina, J. 2006 Comparing the management effectiveness of a marine park and a multiple-use collaborative fisheries management area in East Africa. Aquatic Conservation-Marine and Freshwater Ecosystems 16, 147-165.

[12] Moore, S.A. & Walker, M. 2008 Progressing the Evaluation of Management Effectiveness for Protected Areas: Two Australian Case Studies. Journal of Environmental Policy & Planning 10, 405-421.

[13] Muthiga, N.A. 2009 Evaluating the effectiveness of management of the Malindi-Watamu marine protected area complex in Kenya. Ocean Coast. Manage. 52, 417-423.

[14] Ohnesorge, B., Plieninger, T. & Hostert, P. 2013 Management Effectiveness and Land Cover Change in Dynamic Cultural Landscapes-Assessing a Central European Biosphere Reserve. Ecol. Soc. 18. (doi:10.5751/es-05888-180423).

[15] Parr, C.L., Woinarski, J.C.Z. & Pienaar, D.J. 2009 Cornerstones of biodiversity conservation? Comparing the management effectiveness of Kruger and Kakadu National Parks, two key savanna reserves. Biodivers. Conserv. 18, 3643-3662. (doi:10.1007/s10531-009-9669-4).

[16] Pressey, R.L., Whish, G.L., Barrett, T.W. & Watts, M.E. 2002 Effectiveness of protected areas in north-eastern New South Wales: recent trends in six measures. Biol. Conserv. 106, 57-69. (doi:Doi: 10.1016/s0006-3207(01)00229-4).

[17] Quan, J., Ouyang, Z.Y., Xu, W.H. & Miao, H. 2011 Assessment of the effectiveness of nature reserve management in China. Biodivers. Conserv. 20, 779-792. (doi:10.1007/s10531-010-9978-7).

**S7: Descriptions of the four methodologies given in Table 1 (METT, RAPPAM, EOH, SoP)**

(Taken from Leverington, F., Hocking, H., Pavese, H., Costa, K.L. and Courrau, J. (2008) Management effectiveness evaluation in protected areas – A global study. Supplementary report No.1: Overview of approaches and methodologies. The University of Queensland, Gatton, TNC, WWF, IUCN-WCPA, Australia.)

*Note: Numbers of assessments and countries covered have not been updated to reflect the number of assessments currently held in the GD-PAME.*

## Management Effectiveness Tracking Tool (METT)

*Written with assistance and comments from Sue Stolton*

**Organisation**

World Bank/WWF Alliance

**Primary methodology reference**

Stolton S, Hockings, M, Dudley, N, MacKinnon, K, Whitten, T and Leverington, F (2007) 'Reporting Progress in Protected Areas A Site-Level Management Effectiveness Tracking Tool: second edition.' World Bank/WWF Forest Alliance published by WWF, Gland, Switzerland.

http://www.panda.org/about_wwf/what_we_do/forests/our_solutions/protection/tools/tracking_tool/index.cfm

The Tracking Tool is available in a number of languages.

**Brief description of methodology**

The methodology is a rapid assessment based on a scorecard questionnaire. The scorecard includes all six elements of management identified in the IUCN-WCPA Framework (context, planning, inputs, process, outputs and outcomes), but has an emphasis on context, planning, inputs and processes. It is basic and simple to use, and provides a mechanism for monitoring progress towards more effective management over time. It is used to enable park managers and donors to identify needs, constraints and priority actions to improve the effectiveness of protected area management.

**Purposes**

- ✓ **donor/ treasury evaluation**
- ✓ to improve management (adaptive management)
- ✓ for accountability/ audit

**Objectives and application**

The tool's objectives are stated as:

- ➢ Capable of providing a harmonised reporting system for protected area assessment;
- ➢ Suitable for replication;
- ➢ Able to supply consistent data to allow tracking of progress over time;

- Relatively quick and easy to complete by protected area staff, and thus not reliant on high levels of funding or other resources;
- Easily understood by non-specialists;
- Nested within existing reporting systems to avoid duplication of effort.

(Stolton *et al.* 2007)

The Tracking Tool has been applied in at least 85 countries, primarily by donor agencies and NGOs. It is being used by the World Bank, WWF and the GEF as a mandatory monitoring tool for areas in which they are involved.

'The Tracking Tool has been used to survey the effectiveness of the WWF portfolio of 206 forest protected areas, in Europe, Asia, Africa and Latin America, initially in 2003/4 and then repeated during 2005/6. The World Bank has time series data for project sites in several countries, including Bolivia, India, Philippines, Indonesia and Central Asian republics. The Global Environment Facility (GEF) has adopted the Tracking Tool as a simple impact monitoring indicator, and recently China and India have adopted the tool as part of their national protected area monitoring systems. To aid adoption the tool has been translated into many languages'(MacKinnon and Higgins-Zogib 2006).

The methodology can also be adapted and used by other development programs, protected area management agencies or national governments as a tool to assess protected areas across a group or system, as has been done in Korea (Young 2005) and Namibia (Jonathon Smith *pers. comm.)* and for 150 forest reserves in Tanzania (Neil Burgess *pers. comm.*). An adaptation is also being used in the Brazilian Amazon (Ronaldo Weigand *pers. comm.*).

**Origins**

The World Bank/WWF Alliance for Forest Conservation and Sustainable Use ('the Alliance') was formed in April 1998. As part of its programme of work the Alliance set a target relating to management effectiveness of protected areas: 50 million hectares of existing but highly threatened forest protected areas to be secured under effective management by the year 2005. To evaluate progress towards this target the Alliance developed a simple site-level Tracking Tool to facilitate reporting on management effectiveness of protected areas within WWF and World Bank projects. The Tracking Tool has been built around the application of the IUCN-WCPA Framework.

After being tested and modified over a three-year period, the Tracking Tool has been operational since 2003. A revised version released in 2007 is compatible with the previous version but clarifies some questions and is more consistent in its descriptions of scores.

**Strengths**

The Tracking Tool produces a standard report which has been widely used across the world. It is designed primarily to track progress over time (rather than to compare sites) and can reveal trends, strengths and weaknesses in individual protected areas or in groups. The data set from the Tracking Tool is large enough to reveal some international trends in protected area management (Dudley *et al.* 2004).

It is rapid to complete, with only 30 questions, but covers all the elements of the IUCN-WCPA Framework and, especially if it is applied in a workshop situation, leads to a good deal of discussion and reflection. If it is fully completed, with comments and 'next steps', it can be valuable in setting directions and in evaluating progress towards improving protected area management. '… the Tracking Tool has proven to be a useful instrument to build a baseline on management effectiveness, for tracking progress over time, for providing critical information about portfolio-wide issues that need to be addressed as a priority, and for putting

in place a simple monitoring system in sites that will not afford to develop a more detailed monitoring system in years to come' (MacKinnon and Higgins-Zogib 2006).

**Constraints and weaknesses**

The constraints of the Tracking Tool are acknowledged in its documentation. The assessments produced are relatively superficial (as expected from a rapid analysis) and do not cover all aspects of management. Because of the great differences between expectations, resources and needs around the world, the Tracking Tool is not designed to compare sites.

'The objectives of the Tracking Tool, to be quick and simple, also mean it has limitations as to what it can achieve. It should not, for example, be regarded as an independent assessment, or as the sole basis for adaptive management, and should certainly not replace more thorough methods of assessment for the purposes of adaptive management.' (MacKinnon and Higgins-Zogib 2006). Evaluation of outcomes is not detailed and for this the Tracking Tool should be used in conjunction with other monitoring and evaluation tools.

The experience of some people in the field is that the Tracking Tool is better received by field staff if some additional questions specifically relevant to that area and situation are added.

**How the methodology is implemented**

The Tracking Tool is designed to be simple and implemented with minimal costs. Ideally, the questionnaire should be completed as part of a discussion between, at a minimum, the project officer or task manager, the protected area manager and a representative of local stakeholders. Wider discussions with a number of managers and stakeholders are beneficial where possible. A useful part of the questionnaire for the purpose of project oversight and management improvement is the section on "comments" and 'agreed next steps'.

'The Tracking Tool has been designed to be easily answered by those managing the protected area without any additional research. However, it is useful to review the results of existing monitoring and to spend sufficient time discussing each aspect of management being assessed to arrive at a considered judgement. In most cases, a group of protected area staff from the reserve, project staff or other agency staff should be involved in the assessment; where possible additional external experts, local community leaders or others with knowledge and interest in the area and its management can be involved in completing the assessment' (Stolton *et al.* 2007).

When repeat assessments are undertaken it is advisable to use at least some of the same team members who undertook previous assessments. Where this is not possible the information provided by previous assessors in the text fields of the Tracking Tool will be particularly valuable in guiding the assessment and ensuring consistency in the evaluation being made.

**Elements and indicators**

After introductory questions, 30 questions are asked. The tool has been adapted slightly by different countries and has given rise to other systems including the wetland and marine Tracking Tools. As discussed earlier, some organisations have adapted the Tracking Tool to better suit their needs. It is best if this can be done by adding questions to the end, so that answers to other questions can be analysed in a wider data set if desired.

Note: the indicators shown are from the new version of the Tracking Tool, released in 2007.

**Table 1: Indicators for the Tracking Tool methodology (2007 version)**

| |
|---|
| **Data sheet 1**: Details about the protected area and its management objectives, administration, staffing and funding |

| |
|---|
| **Data sheet 2**: Threat assessment (high, medium, low, not applicable) based on the Conservation Measures Partnership threat hierarchy[1] under the following major headings: |
| 1. Residential and commercial development within a protected area: Threats from human settlements or other non-agricultural land uses with a substantial footprint |
| 2. Agriculture and aquaculture within a protected area: Threats from farming and grazing as a result of agricultural expansion and intensification, including silviculture, mariculture and aquaculture |
| 3. Energy production and mining within a protected area: Threats from production of non-biological resources |
| 4. Transportation and service corridors within a protected area: Threats from long narrow transport corridors and the vehicles that use them including associated wildlife mortality |
| 5. Biological resource use and harm within a protected area: Threats from consumptive use of "wild" biological resources including both deliberate and unintentional harvesting effects; also persecution or control of specific species (note this includes hunting and killing of animals) |
| 6. Human intrusions and disturbance within a protected area: Threats from human activities that alter, destroy or disturb habitats and species associated with non-consumptive uses of biological resources |
| 7. Natural system modifications: Threats from other actions that convert or degrade habitat or change the way the ecosystem functions |
| 8. Invasive and other problematic species and genes: Threats from non-native and native plants, animals, pathogens/microbes or genetic materials that have or are predicted to have harmful effects on biodiversity following introduction, spread and/or increase |
| 9. Pollution entering or generated within protected area: Threats from introduction of exotic and/or excess materials or energy from point and non-point sources |
| 10. Geological events: Geological events may be part of natural disturbance regimes in many ecosystems. But they can be a threat if a species or habitat is damaged and has lost its resilience and is vulnerable to disturbance. Management capacity to respond to some of these changes may be limited. |
| 11. Climate change and severe weather: Threats from long-term climatic changes which may be linked to global warming and other severe climatic/weather events outside of the natural range of variation |
| 12. Specific cultural and social threats |
| **Assessment** |
| 1. Legal status: Does the protected area have legal status (or in the case of private reserves is covered by a covenant or similar)? |
| 2. Protected area regulations: Are appropriate regulations in place to control land use and activities (e.g. hunting)? |
| 3. Law enforcement: Can staff enforce protected area rules well enough? |
| 4. Protected area objectives: Is management undertaken according to agreed objectives? |
| 5. Protected area design: Is the protected area the right size and shape to protect species and habitats of key conservation |
| 6. Protected area boundary demarcation: Is the boundary known and demarcated? |
| 7. Management plan: Is there a management plan and is it being implemented? |

[1] IUCN – Conservation Measures Partnership (2006) IUCN – CMP Unified Classification of Direct Threats Version 1.0 – June 2006. http://www.iucn.org/themes/ssc/sis/classification.htm.

| |
|---|
| 7a. Planning process: The planning process allows adequate opportunity for key stakeholders to influence the management plan |
| 7b. Planning process: There is an established schedule and process for periodic review and updating of the management plan |
| 7c. Planning process: The results of monitoring, research and evaluation are routinely incorporated into planning |
| 8. Regular work plan: Is there a regular work plan and is it being implemented |
| 9. Resource inventory: Do you have enough information to manage the area?<br>10. Protection systems: Are systems in place to control access/resource use in the protected area? |
| 11. Research: Is there a programme of management-orientated survey and research work? |
| 12. Resource management: Is active resource management being undertaken? |
| 13. Staff numbers: Are there enough people employed to manage the protected area? |
| 14. Staff training: Are staff adequately trained to fulfil management objectives? |
| 15. Current budget: Is the current budget sufficient? |
| 16. Security of budget: Is the budget secure? |
| 17. Management of budget: Is the budget managed to meet critical management needs? |
| 18. Equipment: Is equipment sufficient for management needs? |
| 19. Maintenance of equipment: Is equipment adequately maintained? |
| 20. Education and awareness: Is there a planned education programme linked to the objectives and needs? |
| 21. Planning for land use: Does land use planning recognise the protected area and aid the achievement of objectives? |
| 22. State and commercial neighbours: Is there co-operation with adjacent land users? |
| 23. Indigenous people: Do indigenous and traditional peoples resident or regularly using the protected area have input to management decisions? |
| 24. Local communities: Do local communities resident or near the protected area have input to management decisions? |
| 24 a. Impact on communities: There is open communication and trust between local and/or indigenous people, stakeholders and protected area managers |
| 24b. Impact on communities: Programmes to enhance community welfare, while conserving protected area resources, are being implemented |
| 24c. Impact on communities: Local and/or indigenous people actively support the protected area |
| 25. Economic benefit: Is the protected area providing economic benefits to local communities, e.g. income, employment, payment for environmental services? |
| 26. Monitoring and evaluation: Are management activities monitored against performance? |
| 27. Visitor facilities: Are visitor facilities adequate? |
| 28. Commercial tourism operators: Do commercial tour operators contribute to protected area management? |
| 29. Fees: If fees (i.e. entry fees or fines) are applied, do they help protected area management? |
| 30. Condition of values: What is the condition of the important values of the protected area? |
| 30a: Condition of values: The assessment of the condition of values is based on research and/or monitoring |
| 30b: Condition of values: Specific management programmes are being implemented to address threats to biodiversity, ecological and cultural values |

| 30c: Condition of values: Activities to maintain key biodiversity, ecological and cultural values are a routine part of park management |
|---|

**Scoring and analysis**

In the main assessment form, 30 questions are asked - each with a four-point scale (0, 1, 2, and 3). The intention is that the scale forces respondents to choose whether the situation is acceptable or not. Generally 0 is equivalent to no or negligible progress; 1 is some progress; 2 is quite good but has room for improvement; 3 is approaching optimum situation. A series of four alternative answers are provided against each question to help assessors to make judgements as to the level of score given. In addition, there are three groups of supplementary questions which elaborate on key themes in the previous questions and provide additional information and points. Where questions are not relevant to the protected area, they are left out and the scores adjusted accordingly.

The scores are totalled and the percentage of the possible score calculated.

It is noted that 'the whole concept of "scoring" progress is however fraught with difficulties and possibilities for distortion. The current system assumes, for example, that all the questions cover issues of equal weight, whereas this is not necessarily the case. Scores will therefore provide a better assessment of effectiveness if calculated as a percentage for each of the six elements of the IUCN-WCPA Framework (i.e. context, planning, inputs, process, outputs and assessments)' (Stolton *et al.* 2007).
Some analyses have been conducted to discover overall trends and correlations between management strengths and weaknesses. Analyses of repeated surveys have also begun.

**Further reading and reports**

(Dudley *et al.* 2004; Dudley *et al.* 2006; Stolton *et al.* 2003b)

## Rapid Assessment and prioritization of protected area management (RAPPAM)

*Written with assistance and comments from: Alexander Belokurov (WWF) and Jamison Ervin (TNC)*

**Organisation**

WWF

**Primary methodology reference**

Ervin, J. (2003b) WWF: Rapid Assessment and prioritization of Protected Area Management (RAPPAM) Methodology. WWF Gland, Switzerland

WWF (no date) 'Metodología para la evaluación y priorización rápidas del manejo de áreas protegidas (RAPPAM).' WWF.

http://www.panda.org/parkassessment; www.conserveonline/workspaces/patools

**Brief description of methodology**

The RAPPAM methodology is designed for broad-level comparisons among many protected areas which together make a protected areas network or system. It can:

- Identify management strengths, constraints and weaknesses.
- Analyse the scope, severity, prevalence and distribution of threats and pressures.
- Identify areas of high ecological and social importance and vulnerability.
- Indicate the urgency and conservation priority for individual protected areas.
- Help to develop and prioritise appropriate policy interventions and follow-up steps to improve protected area management effectiveness.

It can also answer a number of important questions:

- What are the main threats affecting the protected areas system, and how serious are they?
- How do protected areas compare with one another in terms of infrastructure and management capacity? And how do they compare in effectively producing outputs and conservation outcomes as a result of their management?
- What is the urgency for taking actions in each protected area?
- What are the important management gaps in the PA system?
- How well do national and local policies support effective management of protected areas? Are there gaps in legislation or governance improvements that are needed?
- What are the most strategic interventions to improve the entire system?

*Higgins-Zogib and Lacerda (2006)*

**Purposes**

- ✓ **for prioritisation and resource allocation**
- ✓ to raise awareness and support
- ✓ to improve management (adaptive management) – at system level

**Objectives and application**

RAPPAM provides policy makers and protected area authorities with a relatively quick and easy method to identify major trends and issues that need to be addressed for improving management effectiveness in any given *system* or group of protected areas. Through

conducting RAPPAM assessments, authorities responsible for managing systems of protected areas have been able to:

- analyse the range of major threats facing their protected areas system and to get a broad overview of the most pressing management issues they face;
- look at how the system or group as a whole is functioning and performing; and
- to agree on needed corrective steps that will lead to improved system-level management effectiveness.

RAPPAM has been implemented in some 40 countries and over 1000 protected areas in Europe, Asia, Africa and Latin America and the Caribbean. Useful reports of the status of protected area systems or groups are produced (see list of references at the end of this section), suggesting priority protected areas in terms of the values and vulnerabilities and analysing the trends in protected area management issues.

**Origins**

The system was designed originally to assess networks of protected areas. It is based on the IUCN-WCPA Framework. It was developed by WWF between 1999 and 2002, with field testing in China, France, Cameroon, Algeria and Gabon.

**Strengths**

It has been used widely in different regions of the world and covers network of protected areas in one assessment. It allows identification of threats and management issues across groups of protected areas. In contrast to many other systems, it includes indicators measuring the state of protected area system as a whole, as well as collecting details about individual protected areas.

'A broad-level assessment such as WWF's Rapid Assessment can be complementary to more detailed site-level assessments. It can serve as an early warning for serious management problems, and help identify individual protected areas that may warrant more in-depth study. It can also help identify broad program areas, such as training, PA site design, or law enforcement that may warrant a more thorough analysis and review. Furthermore, a broad-level assessment can be viewed as a type of macro assessment; it can enhance, but is not a substitute for, the routine reviews and evaluations that are part of program planning, implementation and assessment cycles' (WWF 2001).

The workshop looking at MEE in the Andean countries (Cracco et al. 2006)also noted:

- It allows general and comparative evaluations, identifies management strengths and weaknesses, points out the urgency/priority of conservation and provides effective and transparent information for the distribution of resources and the development of policies in the levels of the PA and the country.
- Covers the six elements of the IUCN-WCPA Framework.
- It is easy to adapt.

**Constraints and weaknesses**

The system is not designed to measure outcomes of management in depth. It is primarily designed to assist in setting priorities across a system of protected areas and although it can be applied to a single protected area, the RAPPAM Methodology is not designed to provide detailed, site-level adaptive management guidance to protected area managers.

**How the methodology is implemented**

*The following material has been extracted from Higgins-Zogib and Lacerda (2006)*

'There are five steps in the RAPPAM process:

- Determine the scope of the assessment;
- Assess existing information for each protected area;
- Administer the RAPPAM questionnaire;
- Analyse the findings; and
- Identify next steps and recommendations.

In general the most thorough and effective approach to implementing this methodology is to hold an interactive workshop or series of workshops in which protected area managers, policy makers, and other stakeholders participate fully in evaluating the protected areas, analysing the results and identifying subsequent next steps and priorities.

RAPPAM workshops usually take three days. Two-day workshops have been held, but in these cases the agenda has been very tight with little time available for group and plenary discussions. The costs depend largely on where the workshop is held. Where possible it is advisable to hold the workshop inside a protected area as many of the discussion points during the workshop will be represented right outside the door. However, these logistics are usually the choice of the government ministry (or other protected area authority), who will be the lead player in the workshop.

Getting the right participants to the workshop is critical – and the broader the stakeholder group present, the more true the results. It is important to have at least the manager of each park present at the workshop, as well as top-level participation from the appropriate government ministry. If deemed appropriate, donors can be invited, in the hope that they engage in helping with follow-up steps, as can other international and local NGOs present in the country or region. This helps build support for implementing recommendations that stem from the workshop. Other stakeholders such as community representatives, tourism operators and university staff strengthen the results. And even if in the end, there is disagreement between park staff and community members for example, points raised by the community can still be reflected in the RAPPAM report and taken into consideration.

Lessons learned:

- Ensure the government protected area authority leads the assessment process.
- Develop partnerships with other NGOs present in the country or region.
- Choose a useful assessment scope: RAPPAM is seen at its best when a larger number of protected areas are included in the assessment.
- Administer the questionnaire through interactive workshops.
- Think carefully about assessment objectives and adapt the method to local needs.
- Launch the report at an event if possible.
- Make clear, concrete, practical recommendations.
- Ensure participation and engagement of local communities and other relevant stakeholders in assessments, but plan carefully for their input.

**Elements and indicators**

The questionnaire begins with introductory context questions on values and threats/ vulnerability, followed by questions aimed at the protected area level and the system level. Questions are divided into a number of headings.

**Table 2: Indicators for the RAPPAM methodology**

| WCPA Elements | Sections | Questions |
|---|---|---|
| | 1. Background | includes specific management objectives and critical management activities |
| Context | 2. Pressures and threats | including trend, extent, impact, permanence, and probability of past and future threats |
| Context | 3. Biological importance | Number of rare, threatened or endangered species<br>Relative level of biodiversity<br>Degree of endemism<br>Critical landscape function<br>Extent of full range of plant and animal diversity<br>Contribution to the representativeness of PA system<br>Minimum viable populations of key species<br>Consistency of structural diversity with historic norms<br>Historic range has been greatly diminished ecosystems<br>Extent of full range of natural processes and disturbance regimes |
| Context | 4. Socio-economic importance | Employment for local communities<br>Dependence of communities on PA resources for their subsistence<br>Community development opportunities through sustainable resource use<br>Religious or spiritual significance<br>Unusual aesthetic features<br>Plant species of high social, cultural or economic importance<br>Animal species of high social, cultural or economic importance<br>Recreational value<br>Ecosystem services and benefits to communities<br>Educational and/or scientific value |
| Context | 5. Vulnerability | Low law enforcement<br>Common bribery and corruption<br>Civil unrest and/or instability<br>Conflicting cultural practices, beliefs and traditional uses<br>High market value of PA resources<br>Accessibility for illegal activities<br>Demand for vulnerable resources<br>Pressure to unduly exploit resources<br>Difficult recruitment and retention of employees<br>Difficulty in monitoring illegal activities within the PA |
| Planning | 6. Objectives | PA objectives provide for the protection and maintenance of biodiversity<br>Specific biodiversity-related objectives are clearly stated in the management plan<br>The management policies and plans are consistent with the PA objectives<br>PA employees and administrators understand the PA objectives and policies<br>Local communities support the overall objectives of the PA |

| WCPA Elements | Sections | Questions |
|---|---|---|
| Planning | 7. Legal security | The protected area has long-term legally-binding protection<br>There are no unsettled disputes regarding land tenure or use rights<br>Boundary demarcation is adequate to meet the PA objectives<br>Staff and financial resources are adequate to conduct critical law enforcement activities<br>Conflicts with the local community are resolved fairly and effectively |
| Planning | 8. PA site design and planning | The sitting of the PA is consistent with the PA objectives<br>The layout and configuration of the PA optimises the conservation of biodiversity<br>The PA zoning system is adequate to achieve the PA objectives<br>The land use in the surrounding landscape enables effective PA management<br>The protected area is linked to another area of conserved or protected land |
| Inputs | 9. Staff | The level of staffing is sufficient to effectively manage the area<br>Staff members have adequate skills to conduct critical management activities<br>Training and development opportunities are appropriate to the needs of the staff<br>Staff performance and progress on targets are periodically reviewed<br>Staff employment conditions are sufficient to retain high-quality staff |
| Inputs | 10. Communication and information inputs | There are adequate means of communication between field and office staff<br>Existing ecological and socio-economic data are adequate for management planning<br>There are adequate means of collecting new data<br>There are adequate systems for processing and analysing data<br>There is effective communication with local communities |
| Inputs | 11. Infrastructure | Transportation infrastructure is adequate to perform critical management activities<br>Field equipment is adequate to perform critical management activities<br>Staff facilities are adequate to perform critical management activities<br>Maintenance and care of equipment is adequate to ensure long-term use<br>Visitor facilities are appropriate to the level of visitor use |

| WCPA Elements | Sections | Questions |
|---|---|---|
| Inputs | 12. Finances | Funding in the past 5 years has been adequate to conduct critical management activities<br>Funding for the next 5 years is adequate to conduct critical management activities<br>Financial management practices enable efficient and effective PA management<br>The allocation of expenditures is appropriate to PA priorities and objectives<br>The long-term financial outlook for the PA is stable |
| Process | 13. Management planning | There is a comprehensive, relatively recent written management plan<br>There is a comprehensive inventory of natural and cultural resources<br>There is an analysis of, and strategy for addressing, PA threats and pressures<br>A detailed work plan identifies specific targets for achieving management objectives<br>The results of research and monitoring are routinely incorporated into planning |
| Process | 14. Management decision-making practices | There is clear internal organisation<br>Management decision making is transparent<br>PA staff regularly collaborate with partners, local communities and other organisations<br>Local communities participate in decisions that affect them<br>There is effective communication between all levels of PA staff and administration |
| Process | 15. Research, monitoring, and evaluation | The impact of legal and illegal uses of the PA are accurately monitored and recorded<br>Research on key ecological issues is consistent with the needs of the PA<br>Research on key social issues is consistent with the needs of the PA<br>PA staff members have regular access to recent scientific research and advice<br>Critical research and monitoring needs are identified and prioritised |
| Outputs | 16. Outputs | Threat prevention, detection and enforcement<br>Site restoration and mitigation efforts<br>Wildlife or habitat management<br>Community outreach and educational efforts<br>Visitor and tourist management<br>Infrastructure development<br>Management planning and inventorying<br>Staff monitoring, supervision and evaluation<br>Staff training and development<br>Research and monitoring outputs |

| WCPA Elements | Sections | Questions |
|---|---|---|
| System-level questions | 17. Protected area system design | The PA system adequately represents the full diversity of ecosystems within the region<br>The PA system adequately protects against the extinction or extirpation of any species<br>The PA system consists primarily of exemplary and intact ecosystems<br>Sites of high conservation value for key species are systematically protected<br>The PA system maintains natural processes at a landscape level<br>The PA system includes the protection of transition areas between ecosystems<br>The PA system includes the full range of successional diversity<br>Sites of high biodiversity are systematically protected<br>Sites of high endemism are systematically protected<br>The layout and configuration of the PA system optimises the conservation of biodiversity |
| System-level questions | 18. Protected area policies | National PA policies clearly articulate a vision, goals and objectives for the PA system. The area of land protected is adequate to maintain natural processes at a landscape level<br>There is a demonstrated commitment to protecting a viable and representative PA network<br>There is a comprehensive inventory of the biological diversity throughout the region<br>There is an assessment of the historical range of variability of ecosystem types in the region<br>There are restoration targets for underrepresented and/or greatly diminished ecosystems<br>There is ongoing research on critical PA-related issues<br>The PA system is periodically reviewed for gaps and weaknesses (e.g. gap analyses)<br>There is an effective training and capacity-building programme for PA staff<br>PA management, including management effectiveness, is routinely evaluated |

| WCPA Elements | Sections | Questions |
|---|---|---|
| System-level questions | 19. Policy environment | PA-related laws complement PA objectives and promote management effectiveness<br>There is sufficient commitment and funding to effectively administer the PA system<br>Environmental protection goals are incorporated into all aspects of policy development<br>There is a high degree of communication between natural resource departments<br>There is effective enforcement of PA-related laws and ordinances at all levels<br>National policies promote widespread environmental education at all levels<br>National policies promote sustainable land management.<br>National policies promote an array of land conservation mechanisms<br>There is adequate environmental training for governmental employees at all levels<br>National policies foster dialogue and participation with civic and environmental NGOs |

**Scoring and analysis**

Most questions use a standard 4-selection scale (no=0, mostly no=1, mostly yes=3, yes=5), where ‘yes’ describes an ideal situation. Threats (vulnerability) are rated according to their extent, impact and trend.

Analysis of the data is usually presented as comparisons among the sites in the protected area system. Many different analyses are presented in the reports. Important outputs include lists and graphs of the most common threats, management strengths and management weaknesses; prioritisation of parks with respect to their vulnerability and importance; and other comparative information about specific aspects of management.

**Further reading and reports**

See reference list for full referencing of the following reports in the bibliography or refer to the WWF Website:

(Anonymous no date; Department of Forests and Wildlife Sikkim and WWF India 2003; Diqiang *et al.* 2003; Duguman 2006; Ervin 2003a; Ervin 2004a; b; Goodman 2003; Higgins-Zogib 2004; Higgins-Zogib and Lacerda 2006; Instituto Brasileiro do Meio Ambiente e dos Recursos Naturais Renováveis and WWF-Brasil 2007; Lacerda *et al.* 2004; Ministry of Natural Resources and the Environment 2006; Nemekhjargal and Belokurov 2005; Nepali 2006; Simões 2005; Simoes and Numa de Oliveria 2003; Stanciu and Steindlegger 2006; Steindlegger and Kalem 2005; Tacón *et al.* 2005; Tshering 2003; Tyrlyshkin *et al.* 2003; WWF 2001; 2004; no date; WWF India 2006)

## Enhancing our Heritage

*Written with assistance/comments from Sue Stolton*

**Organisation**

UNESCO, IUCN, and the University of Queensland

**Primary reference**

Hockings M, Stolton, S, Courrau, J, Dudley, N, Parrish, J, James, R, Mathur, V and Makombo, J (2007) 'The World Heritage Management Effectiveness Workbook: 2007 Edition.' UNESCO, IUCN, University of Queensland, The Nature Conservancy.

Hockings, M., Stolton, S., Courrau, J., Dudley, N., Parrish, J., James, R., Mathur, V. and Makombo, J. (2007) 'Libro de trabajo para la efectividad del manejo del Patrimonio Mundial: Edición 2007: 2007 Edition.' UNESCO, IUCN, University of Queensland, The Nature Conservancy.

Available online at www.enhancingheritage.net

**Purposes**

- ✓ **to improve management (adaptive management)**
- ✓ to raise awareness and support
- ✓ for accountability/ audit
- ✓ for prioritisation and resource allocation
- ✓ As this is a toolkit, it can be adapted for multiple purposes

**Brief description of methodology**

The Enhancing our Heritage (EoH) project is developing and testing a toolkit of methodologies, detailed in the *World Heritage Management Effectiveness Workbook* (Hockings *et al.* 2007), which help managers and stakeholders assess current activities, identify gaps and discuss how problems might be addressed. The IUCN-WCPA Framework is the unifying theme around which the Workbook is structured. Indicators and tools for assessing each component of the Framework are suggested to build up a picture of the adequacy and appropriateness of management and the extent to which objectives are being achieved.

The workbook includes 12 tools (see the indicator list) which are based on a variety of best practices in protected area, and in particular World Heritage, assessment. The assessment tools centre on identifying the main values (biodiversity, social, economic and cultural) which the World Heritage Site was set up to protect (and other important values), ensuring that appropriate objectives based on these values have been set, and then assessing the effectiveness of management in achieving these objectives.

Important values are used because, just as it is impossible to manage every species, hectare or social interaction in a protected area, it is impossible to monitor and assess everything that happens there. World Heritage sites vary in their objectives, management approaches, and capacity for assessment and monitoring; so various different tools are provided. The assessment tools can be used either to supplement existing assessment activities, helping to ensure all components of the management cycle are assessed, or to build a complete assessment system from the start' (Hockings *et al.* 2004). The scale and detail of the assessment are likely to vary, depending on available financial and human resources.

**Objectives and application**

The objectives of EOH are to provide site managers and stakeholders with a tested set of tools for developing and implementing a site-based management effectiveness monitoring and evaluation system which:

- focuses on the most important values and objectives of the site;
- addresses key threats to these values and objectives;
- is flexible and enables incorporation of existing monitoring and assessment systems into the overall evaluation; and
- provides for in-depth participatory assessment of important aspects of management for all six of the IUCN-WCPA Framework elements (context, planning, inputs, processes, outputs and outcomes) but pays particular attention to assessing outcomes of management.

It is also valuable for donor/ treasury evaluation, especially to improve the comprehensiveness and usefulness of reporting to the World Heritage Committee.

The EoH methodology is being designed for World Heritage Sites but it has proven to be applicable to other protected areas. 'The UNESCO/IUCN *Enhancing our Heritage* (EoH) project, funded by the United Nations Foundation, is aiming to improve monitoring and evaluation in natural World Heritage sites. The project team, from Europe and Latin America and managed by the University of Queensland, Australia, is working with staff and partners in nine pilot World Heritage sites in Africa, Asia and Latin America to develop and test management assessment methods'(Stolton *et al.* 2006).

Projects currently in development will increase the application of this methodology through awareness raising and capacity building at national and regional levels, training for regionally-based mentors to help guide evaluations and support for extending application of the system to a wider range of countries and sites.

**Origins**

'The EOH project has been in progress since 2001 and the first draft of the manual was published in that year. Many of the tools used in the methodology draw from the experiences in Fraser Island World Heritage site, Australia and from a joint WWF and IUCN project to develop assessment methods in Central Africa, in particular at the Dja World Heritage site, Cameroon. Tools for identifying objectives are based on those developed by The Nature Conservancy (TNC) for use in the USA, the Caribbean and Central and South America. The threat assessment also draws on work by TNC and the Biodiversity Support Program. The methodology developed for assessing ecological integrity (an outcome measure) was inspired by existing systems used by Parks Canada, TNC and Kruger National Park in South Africa' (Stolton *et al.* 2006).

The tools in the workbook have been field-tested and revised, in co-operation with managers and partners, in the nine sites participating in the Enhancing our Heritage project. The insights of those using the tools in these sites (which vary greatly biologically and in their size, level of funding and staffing and knowledge base) were incorporated into in the latest draft of the workbook. The final version of the workbook will be published by UNESCO in 2008.

**Strengths**

The approach provides guidance for an integrated in-depth evaluation of all six elements of the IUCN Management Effectiveness Framework. As it uses a number of different 'tools', it is flexible and can be adapted to suit the local situation, needs and level of resources. Other

systems of evaluation, such as questionnaires already developed to assess inputs, processes or context issues, could be fed into this system if desire.

Unlike many other systems, it places emphasis on the measurement of outcomes of management and assists in both the reporting of monitoring activities and in the development of monitoring priorities and procedures.

It encourages stakeholder participation in both the design and evaluation phases and has resulted in some improved communication in the field. The process can result in considerable capacity strengthening

**Constraints and weaknesses**

The EOH methodology is not a simple 'off-the-shelf' methodology and must be adapted to the individual situation. The system as a whole is relatively time-consuming and expensive, and its implementation requires continuing resourcing and some training and assistance.

**How the methodology is implemented**

The implementation process includes the following steps:

- Training for protected area managers;
- Desktop literature surveys, data collection and review;
- Workshops with staff;
- Workshops with stakeholders;
- Compilation of existing monitoring results; and
- Development of enhanced, values-based monitoring program.

The need for partnerships and local capacity building during the process is stressed: 'The underlying premise of the EoH Project is that World Heritage sites undertake assessment of their own management effectiveness. For the self-assessment process to be rigorous it is essential that site managers assemble a team of stakeholder representatives to work with them to develop and support the monitoring and assessment process. …. The project requirement for site implementation teams to undertake the project, who then work with a wider group of stakeholders to develop and ratify the initial assessment, reinforces this need to build strong and coherent local teams to work together to assess management' (Stolton et al., 2006, p.69).

**Elements and indicators**

The workbook provides worksheets for each tool. The worksheets and accompanying text provide indicators for assessment, but sites can adapt these criteria and indicators to suit local circumstances if required.

**Table 3: Indicators for the EOH methodology**

| Tool | Indicators |
|---|---|
| 1. Management values and objectives | Biodiversity values<br>Other natural values<br>Cultural, social and economic values<br>Principal management objectives |
| 2. Identifying threats - stress, source (potential and current), status of threat (area, | Threats to biodiversity<br>Threats to other natural values<br>Threats to cultural and socioeconomic values |

| | |
|---|---|
| intensity, action, urgency of action) | |
| 3. Relationships with stakeholders and partners | Identify all the stakeholders and partners<br>Details of the stakeholder and the issue being assessed<br>Nature of the relationship between this stakeholder and the issue<br>Economic dependency<br>Impacts – Negative impacts<br>Impacts – Positive contributions<br>Willingness to engage<br>Political/Social influence<br>Organisation of stakeholders<br>Opportunities stakeholders/partners have to contribute to management<br>the Level of engagement of the stakeholder/partner<br>Overall adequacy of stakeholder engagement |
| 4. Review of national context | How adequate is the legislation?<br>To what extent is the legislation used/useful?<br>Is the legislation effective?<br>How high does conservation rank relative to other government policies?<br>Does other government policy relevant to this site contradict or undermine conservation policy?<br>Is there a conscious attempt to integrate conservation within other areas of government policy?<br>Are policies implemented i.e. has the necessary legislation been enacted?<br>International conservation conventions and treaties<br>Are these conventions and treaties reflected in national law?<br>How willing is government to fund the World Heritage site?<br>Does government have the capacity to match its willingness?<br>What is the relationship between site level and agency level staff– e.g. money, staff, training, equipment?<br>What proportion of the agency's budget goes to field operations? |
| 5. assessment of management planning | Name of plan; Level of approval of the plan (L,G,A, S/A,D); Year of preparation, likely completion or most recent review; Year specified for next review of plan<br>Comments (comments should concentrate on the adequacy, currency, and integration of the plan with other planning instruments)<br>Does the plan establish a clear understanding of the desired future for the site?<br>Does the plan provide sufficient guidance on the desired future for the site?<br>Does the plan provide for a process of monitoring, review and adjustment?<br>Does the plan provide an adequate and appropriate policy environment?<br>Is the plan integrated/linked to other significant national/regional/sectoral plans?<br>Is the plan based on an adequate and relevant information base?<br>Does the plan address the primary issues?<br>Are the objectives and actions specified in the plan represented as adequate and appropriate response to the issues?<br>Does the plan take account of the needs and interests of local and indigenous communities? |

| | |
|---|---|
| | Does the plan take account of the needs and interests of other stakeholders?<br>Does the plan provide adequate direction on management actions?<br>Does the plan identify the priorities? |
| 6. Design assessment | List objectives for biodiversity and other natural values<br>Key habitats<br>Size<br>External interactions<br>Connectivity<br>List community objectives for cultural, social and economic values<br>Key area<br>legal status and tenure<br>List management issues related to legal status, access and boundary issues with neighbours<br>Legal status and tenure<br>Access points<br>Neighbours |
| 7. Management needs | Assessing management needs<br>Assessing whether the inputs available match the management needs |
| 8. Assessment of management processes | Management planning: Is there a plan and is it being implemented?<br>Planning systems: Are the planning systems appropriate i.e. participation, consultation, review and updating?<br>Regular work plans: Are there annual work plans or other planning tools?<br>Maintenance of equipment: Is equipment adequately maintained?<br>Management staff facilities: Are the available facilities suitable for the management requirements of the site?<br>Staff/management communication: Do staff have the opportunity to feed into management decisions?<br>Staff training: Are staff adequately trained?<br>Personnel management: How well are staff managed?<br>Financial management: Does the financial management system meet the Critical management needs?<br>Managing resources: Are there management mechanisms in place to control inappropriate land uses and activities (e.g. poaching)?<br>Law enforcement: do staff have the capacity to enforce legislation?<br>Monitoring and assessment: Are management activities monitored against performance?<br>Resource inventory: Is there enough information to manage the World Heritage site?<br>Research: Is there a programme of management- orientated survey and research work?<br>Reporting: Are all the reporting requirements of the World Heritage site fulfilled?<br>Ecosystems and species: Is the biodiversity of the World Heritage site adequately managed?<br>Cultural/ historical resource management: Are the site's cultural resources adequately managed?<br>Are visitor facilities (for tourists, pilgrims etc) adequate?<br>Do commercial tour operators contribute to protected area management? |

| | |
|---|---|
| | Have plans been developed to provide visitors with the most appropriate access and diversity of experience when visiting the World Heritage site?<br>Is there a planned education programme?<br>Access Is visitor access sufficiently controlled?<br>Local communities Do local communities resident in or near the World Heritage site have input to management decisions?<br>Indigenous people Do indigenous and traditional peoples resident in or regularly using the site have input to management decisions?<br>Local, peoples welfare Are there programmes developed by the World Heritage managers which consider local people's welfare whilst conserving the sites resources?<br>State and commercial neighbours: Is there cooperation with neighbouring land/sea users?<br>Conflict resolution: If conflicts between the World Heritage site and stakeholders arise, are mechanisms in place to help find solutions? |
| 9. Assessment of management plan implementation | Achievement of management plan actions |
| 10. Output assessment | Numbers of users (e.g. numbers of visitors, numbers of people using a service, numbers of inquiries answered)<br>Volume of work output (e.g. numbers of meetings held with local communities, number of patrols undertaken, extent of area surveyed in a research programme, numbers of prosecutions instigated)<br>Physical outputs (e.g. length of site boundary delineated and marked, numbers of brochures produced or distributed, number and value of development projects completed) |
| 11. Outcomes of management (suggested) | Size of protected area<br>Ecosystem functioning<br>Renewal of ecosystem<br>Uniqueness<br>Diversity<br>Human well-being<br>Cultural values<br>Recreation management objectives<br>Economic objectives<br>Stresses |
| 12. Achievement of principal objectives | |

**Scoring and analysis**

Many of the indicators in the workbook use a four-point scale. In many of these, a description is provided for each of these levels. However, other questions have qualitative and descriptive answers only, or yes/no answers. As this is a toolkit rather than a definitive system, other systems of scoring and analysis could be fed into different aspects if desired.

Outcome indicators depend on data from monitoring programs and are reported in quantitative terms against nominated target conditions, in a system similar to that used by Parks Canada and the TNC CAP methodology.

Reports are prepared structured around the results from the 12 assessment tools with additional commentary, supporting information and analysis as required. Reports are designed to identify any corrective actions or other responses to the evaluation findings. The goals are to use results for adaptive management measures.

### Further reading and reports

(Dudley and Stolton 2003; GEF ; Hockings *et al.* 2004; Stolton *et al.* 2006; Stolton *et al.* 2003a). See site reports on http://www.enhancingheritage.net/

## NSW State of Parks (Australia)

**Organisation**

NSW Department of Environment and Conservation and the University of Queensland

**Primary references**

Hockings, M., Carter, R.W., Cook, C. and James, R. (in prep.) Accountability, Reporting or Management Improvement? Development of a State of the Parks Assessment System in New South Wales, Australia.

NSW National Parks and Wildlife Service (2005) State of the Parks Proforma and Guidelines. NSW Department of Environment and Conservation.

**Purposes**

- **to improve management (adaptive management)**
- to raise awareness and support
- for accountability/ audit
- for prioritisation and resource allocation
- to support budget submissions to government for increased funding.

**Brief description of methodology**

The methodology consists of a proforma which addresses each of the six elements of the IUCN-WCPA Framework. The proforma is designed to be completed for all or most protected areas in a system to provide data for compilation of a State of the Parks report. It is designed to be completed by small groups of staff involved in the management of each protected area in a small workshop setting. Assessments can be completed on a periodic basis (annually or every 2-3 years). Results from assessments can be used to track progress in individual sites over time, or analysed across a group of parks or the entire park system to provide data relevant to planning and decision making. Results across the entire park system can be used to develop a periodic State of the Parks report.

The proforma consists of four sections incorporating both quantitative and qualitative assessment items. Part A covers descriptive information about each reserve such as size, location, legal designation, IUCN Protected Area Category designation, and relevant legal and contextual information such as designation under international agreements (e.g. World Heritage or Ramsar Conventions). Part B compiles information on staff time and financial inputs into management of each reserve. Part C collects information on the existence and status of a plan of management and other plans (e.g. reserve or regional weed or fire management plans) that helped to direct management of the reserve and identifies the most important reserve values, most significant threats and key stakeholder groups and issues. Part D contains 30 assessment items that required staff to rate performance in a variety of aspects of park management against a four level ordinal scale. In all cases, where a qualitative assessment was required from staff, a justification for the assessment given and/or the sources of information used in making the assessment is required (NSW National Parks and Wildlife Service 2005).

**Objectives and application**

The NSW SoP system is designed to provide an overview of management effectiveness in parks and to identify factors that influence conservation outcomes on parks. The SoP system aims to:

- improve the understanding of the condition of and pressures on the parks system;

- evaluate the effectiveness of management activities against objectives and planned outcomes;
- inform planning and decision-making at all levels of management from statewide to the park level, leading to more effective management;
- act as an induction resource for staff new to a park;
- assist in the allocation of funding and resources; and
- promote effective communication of our management performance to communities (i.e. through the State of the Parks report).

**Origins**

The starting point for the design of the system was a review of existing management effectiveness evaluation systems around the world, focusing particularly on those systems that had been designed using the IUCN-WCPA Framework. The basic structure of the NSW State of the Parks system was built around adaptations of components taken from:

- the World Bank/WWF Alliance Management Effectiveness Tracking Tool (Stolton et al 2003), for identification of reserve values and qualitative assessment of management performance;
- WWF Rapid Assessment and Prioritisation of Protected Area Management (Ervin 2003), for assessment of threats; and
- UNESCO/IUCN Enhancing our Heritage Workbook (Hockings et al. 2001).

Additional features were added to improve the reliability and credibility of the staff assessments. These additions particularly focused on providing additional justification for assessments and documentation of sources of information used in making assessments.

**Strengths**

The NSW SoP system provides a relatively rapid and comprehensive methodology for assessing effectiveness of management for large numbers of protected areas. It has been applied to over 700 reserves in New South Wales. It provides information to support adaptive management, planning and decision making at the site level, across regional groups of reserves or across and entire system of protected areas. It also provides information for accountability and reporting at a system level through State of the Parks reporting.

It is designed to provide a consistent assessment across a system of protected areas in which individual parks may have very different levels of underlying monitoring data available to support the assessment. It combines quantitative data and qualitative assessments with the qualitative assessments being justified and supported by monitoring data where this is available. It addresses all six elements of the IUCN-WCPA Framework. Data can be analysed across the park system to identify key factors influencing management effectiveness.

The methodology is readily adaptable to other protected area systems and the can be used with the approval of the NSW Department of Environment and Conservation.

**Constraints and weaknesses**

The qualitative assessment items may vary in reliability depending on the knowledge and training of staff completing the assessment. This methodology shares this limitation with almost all other assessment systems (except those few, resource-intensive systems that rely on quantitative data). However, the NSW SoP system goes to considerable lengths to minimise any possible bias with extensive guidelines and collection of justification information. It also

identifies underlying monitoring or research data used to support assessments where this is available.

**How the method is implemented**

The proforma has been used as both an Excel spreadsheet and as an online web-based form. The following method has been used in NSW and is recommended for any adaptation of the evaluation method to another protected area system (Hockings *et al.* in prep.):

1. Workshop indicators with staff to ensure that the methodology covers the most important aspects of management for the system being assessed and that the indicators reflect appropriate performance standards for the agency.
2. Revise indicators and guidance notes (if necessary) based on the results of the workshop(s).
3. Train staff in application of the methodology.
4. Assemble relevant information for each site in preparation for the assessment (budget information, results of monitoring programs being conducted in the protected area etc).
5. Conduct assessments for each protected area using a small working meeting of key staff involved in and other knowledgeable people (working session to complete the assessment normally lasts one day).
6. Compile and analyse results across the system of protected areas.
7. Feedback results to the protected area agency staff.
8. Periodically prepare State of Parks report (perhaps every 5-6 years).

**Elements and indicators**

Criteria and indicators are classified according the WCPA elements as well as their functional area.

**Table 4: Indicators for NSW SoP methodology**

| WCPA Element | Criteria | Indicator |
|---|---|---|
| Context | Values | Top 5 values for which park is managed |
| | | Significance of values (international, national or local) |
| | Threats | Top 5 current threats to values with assessment of impact and extent of threat |
| | | Top 5 emerging threats to values with assessment of likely impact and extent of threat |
| | Stakeholders | Five primary stakeholders/issues with assessment of the nature of relationship between agency and stakeholder group |
| Planning | Plan of management | Existence and age of plan of management |
| | Subsidiary plans | Type, status, age and influence on management of other plans (e.g. fire management invasive species, visitor management) |
| | Planning and decision making | Identification and use of reserve values in management decision making |
| | | Existence of clear management directions |

| WCPA Element | Criteria | Indicator |
|---|---|---|
| Inputs | Budget | Recurrent budget by function (various aspects of natural resource management, visitor management, cultural heritage management administration etc) |
| | | Capital works budget by function (various aspects of natural resource management, visitor management, cultural heritage management administration etc) |
| | | Revenue raised from park user and other fees |
| Inputs | Staff time and other labour inputs | Staff time and other labour input by function (natural resource management, visitor management, cultural heritage management, administration etc) |
| | Information availability | Adequacy of natural resource information to support decision making |
| | | Adequacy of historic heritage information to support decision making |
| | | Adequacy of indigenous heritage information to support decision making |
| | | Adequacy of information about park visitors to support decision making |
| Processes | Natural resource management | Existence and adequacy of planned approach to weed management |
| | | Existence and adequacy of planned approach to pest animal management |
| | | Existence and adequacy of planned approach to fire management (in relation to both natural and cultural resources) |
| | | Existence and adequacy of planned approach to threatened species management |
| | Social/cultural management | Existence and adequacy of planned approach to visitor impact management |
| | | Existence and adequacy of planned approach to indigenous heritage management |
| | | Existence and adequacy of planned approach to historic heritage management |
| | Law enforcement | Existence and adequacy of planned approach to law enforcement |
| | Infrastructure/asset maintenance | Adequacy of maintenance program |
| | Consultation with stakeholders | Adequacy of consultation processes with indigenous communities |
| | | Adequacy of consultation processes with local and general communities |
| | Monitoring | Existence of a planned approach to monitoring and evaluation |
| Outputs | | Visitor numbers |

| WCPA Element | Criteria | Indicator |
|---|---|---|
| | Visitors, visitor facilities and information | Adequacy and appropriateness of visitor facilities |
| | | Adequacy and appropriateness of visitor information and signage |
| | | Existence and adequacy of planned approach to interpretation and visitor awareness/education |
| | Implementation of plans and work programs | Extent of implementation of management directions |
| | | Existence and extent of implementation of work program for park |
| Outcomes | Natural resource management | Extent to which weed impacts on park values are being controlled |
| | | Extent to which pest animal impacts on park values are being controlled |
| | | Extent to which fire is being managed to meet ecological and cultural heritage management objectives for park |
| | | Condition of threatened species in park |
| | | Condition of nominated principal natural resource values |
| | | Whether change in condition of natural resource values can be attributed to management actions |
| | | Condition of nominated wilderness resource values |
| | | Whether change in condition of wilderness resource values can be attributed to management actions |
| | Social/cultural management | Extent to which visitor impacts on park values are being controlled |
| | | Extent to which impacts on indigenous heritage values are being controlled |
| | | Extent to which impacts on cultural heritage values are being controlled |
| | | Condition of nominated principal indigenous heritage values |
| | | Whether change in condition of indigenous heritage values can be attributed to management actions |
| | | Condition of nominated principal historic heritage values |
| | | Whether change in condition of historic heritage values can be attributed to management actions |
| | Other values | Condition of other nominated park values |

| WCPA Element | Criteria | Indicator |
|---|---|---|
| | | Whether change in condition of other park values can be attributed to management actions |
| | Law enforcement | Extent to which impacts of illegal activities on park values are being controlled |
| | Visitor information | Extent to which visitor and information needs are being met through awareness/education programs |

**Scoring and analysis**

The methodology uses a mix of quantitative and qualitative indicators. Most qualitative indicators are scored on a four point ordinal descriptive scale. In addition to the rating on this scale, information is collected on the justification for the rating that is given, the sources of information used in making the assessment, the proposed actions to be taken in relation to the issue over the coming twelve months, and the extent to which actions for the previous twelve months had been achieved.

Analysis can be conducted on individual sites or, more commonly, on groups of sites or the whole system of protected areas. Performance can be reported on a site or area basis (i.e. the number of sites performing at a specific level or the percentage area of the total estate in different performance categories. Correlation and pattern analysis across a dataset for a protected area system can identify possible factors influencing park management performance.

## Victorian State of Parks (Australia)

**Organisation**

Parks Victoria, Australia.

**Purposes**

- ✓ **to improve management (adaptive management)**
- ✓ to raise awareness and support
- ✓ for accountability/ audit
- ✓ for prioritisation and resource allocation
- ✓ to support budget submissions to government for increased funding.

**Brief description of methodology**

This methodology was developed to provide information to prepare a State of the Parks report. The methodology consists of a proforma that addresses each of the six elements of the IUCN WCPA Management Effectiveness Framework. The proforma is designed to be completed for all or most protected areas in a system to provide data for compilation of a State of the Parks report. It is designed to be completed by park staff in a workshop setting under the direction of a trained facilitator. A single workshop covers a group of geographically related parks. Results from assessments can be used to track progress in individual sites over time, or analysed across a group of parks or the entire park system to provide data relevant to planning and decision making.

**Objectives and application**

The Parks Victoria SoP system is designed to provide an overview of management effectiveness in parks and to identify factors that influence conservation outcomes on parks. The SoP system aims to:

- promote effective communication of our management performance to communities (i.e. through the State of the Parks report )
- improve the understanding of the condition of and pressures on the parks system;
- evaluate the effectiveness of management activities against objectives and planned outcomes; and
- inform planning and decision-making at all levels of management from statewide to the park level, leading to more effective management.

**Origins**

The system has developed from an original State of the Parks report prepared in 2000 that was a more descriptive account of the park system and pressures on individual reserves. Additional elements have been added to incorporate data from existing research and monitoring programs and agency databases. Qualitative assessment items were incorporated based on items from the NSW State of the Parks methodology.

**Strengths**

The Parks Victoria SoP system provides a relatively comprehensive methodology for assessing effectiveness of management for large numbers of protected areas. Incorporation of data from diverse sources means that it is less rapid than the NSW State of the Parks system. It has been applied to 400 reserves in Victoria. It provides information to support adaptive management, planning and decision making at the site level, across regional groups of reserves or across and entire system of protected areas. It also provides information for accountability and reporting at a system level through State of the Parks reporting.

It is designed to provide a consistent assessment across a system of protected areas in which individual parks may have very different levels of underlying monitoring data available to support the assessment. It combines considerable quantitative data together with qualitative assessments. Qualitative assessments information is justified and supported by monitoring data where this is available. It addresses all six elements of the IUCN-WCPA Framework. Data can be analysed across the park system to identify key factors influencing management effectiveness.

**Constraints and weaknesses**

The qualitative assessment items may vary in reliability depending on the knowledge and training of staff completing the assessment. This methodology shares this limitation with almost all other assessment systems (except those few, resource-intensive systems that rely on quantitative data). However the Parks Victoria SoP system goes to considerable lengths to minimise any possible bias by using a facilitated workshop to complete assessments and collection of justification information for any ratings. It also identifies underlying monitoring or research data used to support assessments where this is available.

**How the method is implemented**

The proforma is developed as an Access database. The following process was used in applying the system:

1. Train facilitators in application of the methodology.
2. Assemble relevant information for each site in preparation for the assessment (information from agency databases, results from research and monitoring programs being conducted in each protected area etc).
3. Conduct assessments workshops of key staff involved in management of a group of parks in an administrative region.
4. Compile and analyse results across the system of protected areas and incorporate data from corporate databases in
5. Feedback results to the protected area agency staff
6. Prepare State of Parks report (planed for every 5-6 years).

**Elements and indicators**

The elements and indicators are similar to the ones from the NSW State of Parks (see previous section).

**Scoring and analysis**

The methodology uses a mix of quantitative and qualitative indicators. Most qualitative indicators are scored on a four point ordinal descriptive scale. In addition to the rating on this scale, information is collected on the justification for the rating that is given, the sources of information used in making the assessment, the proposed actions to be taken in relation to the issue over the coming twelve months, and the extent to which actions for the previous twelve months had been achieved.

Analysis can be conducted on individual sites or, more commonly, on groups of sites or the whole system of protected areas. Performance can be reported on a site or area basis (i.e. the number of sites performing at a specific level or the percentage area of the total estate in different performance categories. Correlation and pattern analysis across a dataset for a protected area system can identify possible factors influencing park management performance.
